# The hidden therapist: evidence for a central role of music in psychedelic therapy

**DOI:** 10.1007/s00213-017-4820-5

**Published:** 2018-02-02

**Authors:** Mendel Kaelen, Bruna Giribaldi, Jordan Raine, Lisa Evans, Christopher Timmerman, Natalie Rodriguez, Leor Roseman, Amanda Feilding, David Nutt, Robin Carhart-Harris

**Affiliations:** 1 Psychedelic Research Group, Department of Medicine, Imperial College London, London, W12 0NN UK; 2 School of Psychology, Sussex University, Brighton, BN1 9RH UK; 3 Computational, Cognitive and Clinical Neuroscience Laboratory (C3NL), Department of Medicine, Imperial College London, London, W12 0NN UK; 4 The Beckley Foundation, Oxford, OX3 9SY UK

**Keywords:** Psychedelic therapy, Depression, Psilocybin, Music

## Abstract

**Rationale:**

Recent studies have supported the safety and efficacy of psychedelic therapy for mood disorders and addiction. Music is considered an important component in the treatment model, but little empirical research has been done to examine the magnitude and nature of its therapeutic role.

**Objectives:**

The present study assessed the influence of music on the acute experience and clinical outcomes of psychedelic therapy.

**Methods:**

Semi-structured interviews inquired about the different ways in which music influenced the experience of 19 patients undergoing psychedelic therapy with psilocybin for treatment-resistant depression. Interpretative phenomenological analysis was applied to the interview data to identify salient themes. In addition, ratings were given for each patient for the extent to which they expressed “liking,” “resonance” (the music being experienced as “harmonious” with the emotional state of the listener), and “openness” (acceptance of the music-evoked experience).

**Results:**

Analyses of the interviews revealed that the music had both “welcome” and “unwelcome” influences on patients’ subjective experiences. Welcome influences included the evocation of personally meaningful and therapeutically useful emotion and mental imagery, a sense of guidance, openness, and the promotion of calm and a sense of safety. Conversely, unwelcome influences included the evocation of unpleasant emotion and imagery, a sense of being misguided and resistance. Correlation analyses showed that patients’ experience of the music was associated with the occurrence of “mystical experiences” and “insightfulness.” Crucially, the nature of the music experience was significantly predictive of reductions in depression 1 week after psilocybin, whereas general drug intensity was not.

**Conclusions:**

This study indicates that music plays a central therapeutic function in psychedelic therapy.

**Electronic supplementary material:**

The online version of this article (10.1007/s00213-017-4820-5) contains supplementary material, which is available to authorized users.

## Introduction

The capacity of psychedelic drugs to facilitate emotional release, peak- or mystical experiences, and autobiographical insight was a primary motivation for their therapeutic use in the 1950s and 1960s in the form of “psychedelic therapy” (Busch and Johnson 1950). Music was introduced within the therapeutic framework as a way to support patients’ experiences non-verbally (Bonny and Pahnke 1972; Grof 1980; Hoffer 1965) and has thence remained a staple component of the treatment model. Recent clinical trials have rekindled interest in psychedelic therapy (Carhart-Harris and Goodwin 2017), with positive findings for depression (Carhart-Harris et al. 2016a; de Osório et al. 2015), addiction (Bogenschutz et al. 2015; Johnson et al. 2014, 2016), end-of-life care (Gasser et al. 2014; Griffiths et al. 2016; Grob et al. 2011; Ross et al. 2016), and post-traumatic stress disorder (Mithoefer et al. 2011, 2013).

Psychedelic therapy sessions do not adhere to one specific psychotherapeutic model; however, music-listening is a consistent feature. In psychedelic therapy sessions, during drug effects, patients are encouraged to focus their attention inwards while lying down in a relaxed position and listening to a carefully designed music playlist for the duration of the session. In this way, it is believed that music can help facilitate experiences that have therapeutic import. Studies have shown that psychedelics significantly modulate music-evoked emotion (Kaelen et al. 2015, 2017), music-evoked mental imagery (Kaelen et al. 2016), and perceived personal meaningfulness of music (Preller et al. 2017). In addition, patients undergoing psychedelic therapy often refer to music as influencing their experience significantly (Belser et al. 2017; Swift et al. 2017; Watts et al. 2017). Although these studies support the hypothesis that the subjective response to music is intensified under psychedelics, no studies to date have focussed on the therapeutic functions of music in the context of psychedelic therapy. The present study sought to address this knowledge gap by studying the ways music is experienced during psychedelic therapy sessions, and what variables are most influential in driving positive therapeutic outcomes.

We conducted interviews with patient’s who underwent psychedelic therapy for treatment-resistant depression with psilocybin, 1 week after the second of two treatment sessions. In these interviews, we inquired about patients’ experiences with the music heard during their therapy sessions and the specific different ways in which the music influenced their subjective experiences. According to present theories (Bonny and Pahnke 1972), we hypothesised that music would promote so-called mystical experiences^1^ (Maclean et al. 2012; Maslow 1964; Stace 1960), which would subsequently predict long-term therapeutic outcomes. Endorsing this view, both music (Gabrielsson and Wik 2003) and psychedelics (Griffiths et al. 2006) have separately been associated with the facilitation of mystical experiences, and mystical experiences have been associated with positive therapy outcomes to psychedelic therapy (Garcia-Romeu et al. 2014; Griffiths et al. 2016; Roseman et al. 2017; Ross et al. 2016).

## Methods

### Approvals

The National Research Ethics Service London (West London) provided a favourable opinion for this study. The study was sponsored and approved by Imperial College London’s Joint Research and Compliance Office (JRCO), and the National Institute for Health Research Clinical Research Network adopted the study. The National Institute for Health Research/Wellcome Trust Imperial Clinical Research Facility provided approval for the study site. The study was performed in accordance with the ethical standards laid down in the 1964 Declaration of Helsinki. A Home Office Licence for storing and dispensing Schedule One drugs was obtained.

### Participants

Nineteenpatients with treatment-resistant major depressive disorder where included in the study. Inclusion criteria for the study were moderate to severe major depression, as determined by a score of 17 or higher on the 21-item Hamilton Depression Rating scale (HAM-D), with absence of improvements despite at least two different pharmaceutical antidepressant treatments for a minimum of 6 weeks within the current depressive episode. Exclusion criteria included current or previously diagnosed psychotic disorder, diagnoses of psychotic disorders in immediate family members, history of suicide attempts that required hospitalisation, history of mania, having a blood or needle phobia, pregnancy, and current drug or alcohol dependence.

### Experiment overview and procedures

This study was part of a larger study assessing safety and efficacy for using psilocybin to treat depression (Carhart-Harris et al. 2016a). Psilocybin was synthesised and obtained from THC-Pharm (Frankfurt, Germany) and formulated into 5-mg capsules of psilocybin, by Guy’s and St. Thomas’ Hospital’s Pharmacy Manufacturing Unit (London, UK). Screening consisted of evaluating the patient’s current and past physical and mental health. The 16-item Quick Inventory of Depressive Symptoms (QIDS) patient-rated scale for the severity of depressive symptoms was completed during the screening visit and served as baseline- and post-treatment measure. Written informed consent was obtained from patients, and by the end of the screening, eligible patients met with the two therapists that would support them through the remainder of the trial.

A subsequent visit functioned as preparation for the session. This included conversations with the therapists about the patient’s personal history, expectations for the sessions, and education about the effects of psilocybin. The patient also had an opportunity to listen to samples of session music while wearing eye-shades, as a simulation experience in preparation for their first session. The preparation visit lasted approximately 4 h in total. Following the preparation session, patients received two different dosages of psilocybin on two separate subsequent occasions, each separated by 1 week. In the first session, all patients received an oral dose of 10-mg psilocybin. This lower dose was intended to function like a “taster,” a preparation for the higher dose administered 1 week later. In the second session, all patients received 25 mg. Prior work demonstrated that high doses of psilocybin are linked with greater positive behaviour changes in healthy volunteers (one month prior sessions), when participants received an ascending sequence of doses, compared to volunteers who received a high dose first (Griffiths et al. 2011).

Each session included only one patient and two therapists and took place in a specially designed therapeutic environment. Each session started with arrival at the research facility at 9 am, with psilocybin being administered at 10.30 am. The majority of patients were ready to leave the facility approximately 7 h after administration. Transport from the research facility to home was organised ahead of the sessions and consisted of being accompanied by a close friend or relative. Patients also had the option of staying overnight in accommodation adjacent to the research facility, the night before and the night after the session.

Clinical improvement was defined as reductions in depression severity, measured via the QIDS, completed by all patients at baseline and 1 week after the second and final psilocybin session. The different aspects of the subjective experience of psilocybin were measured with the 11-dimensional Altered States of Consciousness Scale (11D-ASC) (Dittrich 1998) at the end the session. After each session, drug intensity was self-rated on a visual analogue scale (VAS). The question was formulated as “How intense were the peak drug effects?”, with the following anchors on the response scale: “0 = no effects,” “100 = most imaginable.” The interview assessing the patient’s experience of the music was always conducted 1 week after the high dose (25 mg) session. For a detailed report on the clinical outcomes see Carhart-Harris et al. (2016a).

### Therapeutic setting

In consideration of the importance of the therapy environment (Johnson et al. 2008), all sessions took place in a specially designed therapy room within the Clinical Research Facility at Imperial College London. All unnecessary medical equipment was either removed or hidden; light quality was adjusted using Philips *livingcolors* and *Imageo* lights. Cushions, plants, art paintings, and artefacts were introduced to engender a cosy and comfortable climate. After receiving psilocybin, patients were encouraged to relax on the bed and wear *Mindfold* eye-shades. Two therapists were present on either side of the bed and “checked in” with the patient approximately every 30–60 min, to obtain insight into how their subjective experience was unfolding and to determine whether psychological support might be needed. Calming ambient music was played on entrance, but the session playlist (see Supplementary material) was started on ingestion of psilocybin. Patients had the option of listening to the music via high quality in-ear headphones (Sennheiser IE 800) or via a high fidelity standing stereo speaker (Meridian DSP3200). Both headphones and speakers received the same audio signal, which allowed the music be played in synchrony and continuously through both channels. This set-up was considered helpful for (1) providing a sense of continuity in case the headphones were abruptly removed or muted, (2) enabling the therapists to empathise with the patient’s current state, as well as observe how they were responding to a particular piece of music, and (3) allowing a deeper immersion in the music and depth of sound.

### Music selection

The central purpose of the use of music in the present therapeutic study was consistent with that of early psychedelic therapy studies, i.e. to facilitate personally meaningful experiences that can lead to sustained changes in behaviour and outlook. In order to achieve this, researchers often emphasised the importance of adapting the music to individual patient’s changing therapeutic needs, as their therapeutic experience unfolds dynamically (Bonny and Pahnke 1972; Grof 1980; Hoffer 1965). For the present study, however, a standardised playlist was created to control for music as a potential confounding variable. Therefore, all patients were intended to listen to the same music playlist. In one rare case where the music selection was strongly disliked by one patient (#6) in the first session (a strong preference was expressed to only listen to classical music), a music playlist used by Johns Hopkins University was used for the second session (Richards 2015), which includes music originally suggested by Bonny and Pahnke (1972).

Several of the musical works originally included in playlists for psychedelictherapy are very familiar today. Examples include “Samuel Barber—Adagio for strings” and “Beethoven—Piano Concerto 5.” Such high familiarity may reduce the opportunity for patients to have a new experience with the music, unfettered by prior associations. In addition, a strong emphasis on music with “Christian religious” content may not be appropriate for individuals that are either non-religious or practice a different religion. Therefore, a music playlist was designed for the present study, containing predominantly contemporary music such as the ambient, neo-classical, contemporary classical, as well as traditional/ethnic music styles. The intention with this music selection was to minimise religious associations and to support mystical experiences within a secular framework.

### Playlist design

The design of the music playlist was informed by Bonny and Pahnke (1972), William Richards (2015), and the psychedelic therapist StanislavGrof (1980), who defined different phases in psychedelic therapy sessions, where each phase is associated with a distinguishable set of psychological needs the music can serve. These phases are, in chronological order: “pre-onset,” “onset,” “building towards peak,” “peak,” “re-entry,” and “return.” In the present study, the durations of the phases were adjusted to the shorter duration of psilocybin’s effects, compared with LSD. Furthermore, onset and building towards peak were grouped together as “ascent,” and re-entry was named “descent.” Music with strong evocative emotional sentiments was only played during peak, on the assumption that an important pre-requisite is for the individual to first feel calm and safe and that more evocative music would enable an activation of autobiographical and therapeutically significant when played at peak (Bonny and Pahnke 1972). See Supplementary material for the full playlist.

### The semi-structured interview

The semi-structured interview was always conducted 1 week after the final session, by the same researcher, apart from on one occasion, and always in reference to the music experience for both sessions. The interview consisted of four open questions: (1)“Did the music influence your experience? And if so: in what ways?”, (2)“Can you comment on how the different styles of music influenced your experience? And which music did you prefer?”, (3)“Were there any aspects in the music that influenced your experience in a positive way?”, and (4)“Were there any aspects in the music that influenced your experience in a negative way?”. Additional questions were sometimes asked to clarify patient’s responses if the interviewer felt the need to do so (for example “Can you explain what you mean with *x*?” or “Can you tell me more about *x*?”).

### Theoretical approach of interview data analysis

Interpretative phenomenological analysis (IPA) was chosen to analyse the interviews (Smith et al. 1997). IPA is an approach increasingly used in healthcare research (Biggerstaff and Thompson 2008; Smith 2011) via which researchers examine the meanings particular experiences have for people. It is particularly appropriate for ascertaining the complexity (i.e. the quality and phenomenology of experience) of patients’ subjective experience of music during the therapeutic sessions and has previously been used to investigate the benefits of music therapy interventions in cancer care settings (Pothoulaki et al. 2012).

### Interview data analysis: coding

Interviews were transcribed verbatim in Microsoft Word and checked for accuracy. A step-by-step coding analysis of the interview data followed. All coding was done by at least two researchers. This was initially done independently, and at later staged compared between researchers and integrated in a final coding for each transcript. All transcripts were first read through twice, before carrying out the coding analysis. During the third reading, any phrases considered pertinent in terms of how the music influenced the patients’ subjective experience were highlighted and coded into initial interpretations in Microsoft Excel.At this stage, sometimes more than one interpretation was assigned when more than one interpretation could be made, and queries about the meaning of what was being said were recorded in a separate column.

Keywords, phrases, and initial interpretations from the first four completed transcripts were then comprehensively explored, leading to the creation of themes in a separate column. These themes were then used as guidelines for subsequent transcripts. As new themes emerged from these subsequent transcripts, all data were iteratively scrutinised and the list of themes refined. Throughout the process of analysis, codes were examined and discussed among the authors to decide which themes were the most accurate reflections of participants’ experiences.

Following further discussion between authors,a final master list of themes emerged. The final stage of analysis involved the organisation of the list of themes into a more concise list of overarchingsuperordinate “clusters” or domains representing the patient experience of musiceach of which contained a number of subsidiary themes. After all transcripts were coded into themes and clusters,the presence of each unique theme and cluster across all patients’ responses was calculated. This lead to a percentage index describing the frequency to which the themes and clusters were present within the total study population

### Ratings for music experience

Three variables that displayed a notable polarity were identified from the coding analysis and were hypothesised as predictors for therapy response: (1) “liking,” referring to the degree to which the music styles and the music quality were liked, (2) “resonance,” referring to the degree to which the music matched with or was “harmonious” with the intrinsic emotional state of the patient, and (3) “openness,” referring to the degree in which the patient was open to, or accepting of the music-evoked experience. The concept of openness has been more generally discussed in prior literature as an important psychological variable in psychedelic therapy (Grof 1980; Richards 2015).

Four researchers that were blind to patient identifiers and treatment outcomes rated these variables independently for all 19 patients based on their interview transcripts, to ensure inter-rating reliability. Ratings were done via a VAS, with five anchors presented. For liking, the question was formulated as “To what extent did the patient like or dislike the music?”, with the following anchors on the response scale: “0 = strong disliking,” “25 = major disliking, some liking,” “50 = mixed disliking and liking,” “75 = major liking, some disliking,” and “100 = major liking.” For resonance, the question was formulated as “To what extent was the music experienced by the patient as in resonance with his/her subjective experience?”, with the following anchors on the response scale: “0 = strong dissonance,” “25 = major dissonance, some resonance,” “50 = mixed resonance and dissonance,” “75 = major resonance, some dissonance,” and “100 = strong resonance.” And finally, for the third, the question was formulated as “To what extent was the patient accepting of or open to the music-evoked experience?”, with the following anchors on the response scale: “0 = strong resistance,” “25 = major resistance, some openness,” “50 = mixed resistance and openness,” “75 = major openness, some resistance,” and “100 = strong openness.”

### Correlation analyses

The average of the scores from all researchers was calculated for each music variable and for each patient. Pearson correlation tests were performed between the three music experience variables and ratings from the 11D-ASC. To reduce the number of comparisons, a principle component analysis (PCA) was performed on the 11 factors of the 11D-ASC. Varimax rotation was performed on the first fiveprincipal components (PCs) that explained over 95% of the variance (see Fig. 3 for rotated PCs and their respective loadings). Subsequently, the three music experience variables (liking, resonance, and openness) were correlated with these five PCs. To test whether music experience and drug intensity are associated differently with different aspects of subjective experience of psilocybin, drug intensity ratings were also correlated with all five PCs.

Pearson correlation tests were applied to test for a relationship between the music experience variables and reductions in depressive symptoms 1 week after the last session with 25-mg psilocybin. Reductions in depressive symptoms were defined as the percentage reduction in scoring on QIDS relative to baseline (i.e. ((baseline score − post-treatment-score) / baseline score) × − 100).To test for the discriminative value of music experience variables in predicting therapy response, compared with mere drug intensity, ratings for drug intensity were also correlated with reductions in depression. To test for differences between music experience and drug intensity effects, ratings for drug intensity were correlated with each music experience value. The three music experience variables were correlated with each other to test for their discriminative value. Inter-rating reliability of researcher’s ratings was tested by correlating ratings of all researchers with each other. False discovery rate (FDR) control was used to correct for multiple comparisons (Benjamini and Hochberg 1995).

## Results

**Fig.1:**
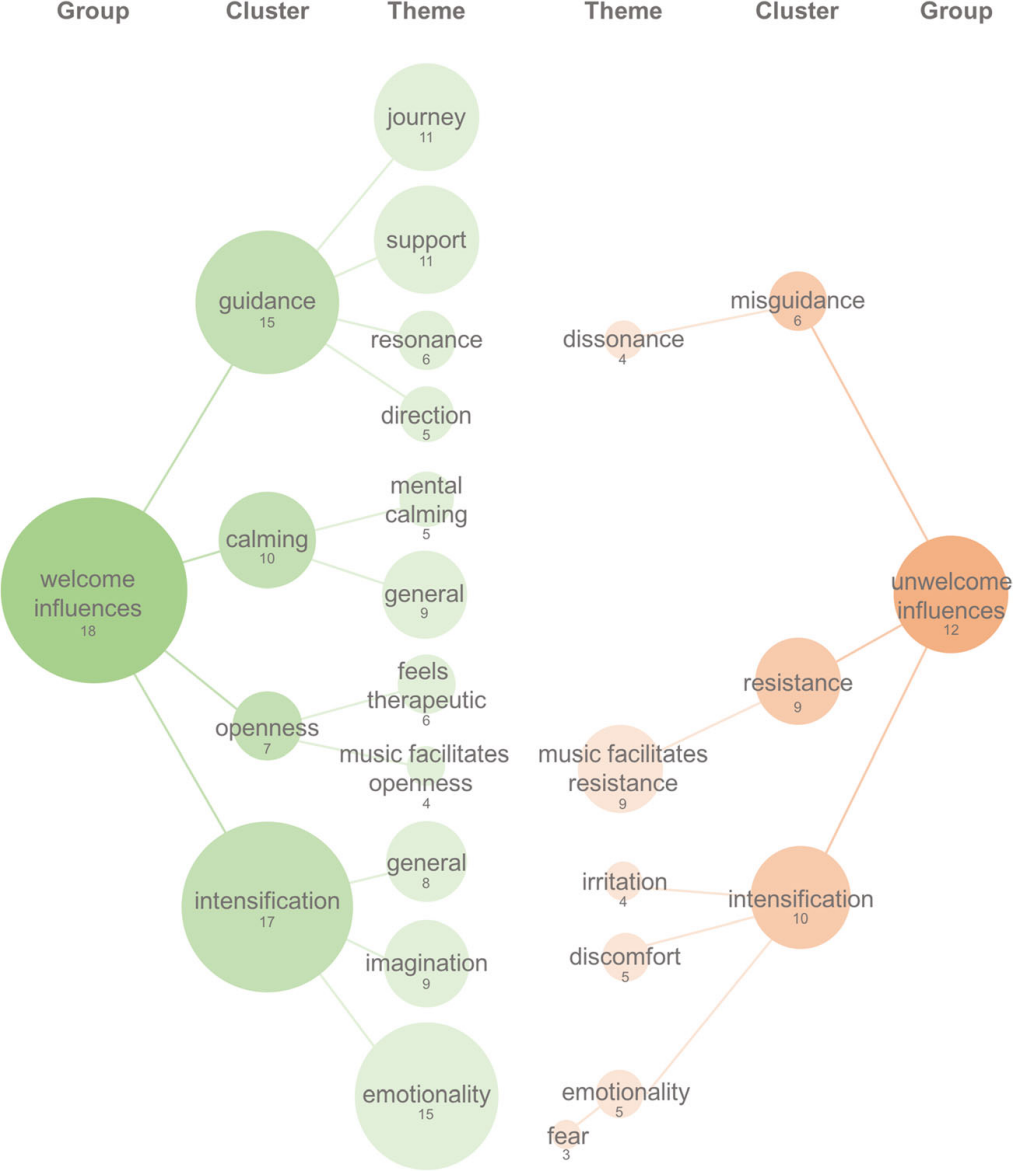
Welcome and unwelcome influences of the music. Welcomed influences are displayed on the left in green, and unwelcomed influences are displayed on the right in red. All clusters and themes that are defined as an accepted or welcomed influence of the music on subjective experience. The figure displays cluster present in more than 30% of all participants and per cluster the themes that were present in more than 30% of the cluster. The numbers below the group-, cluster-, or theme-name refers to the total number of patients that referred to this. The size of the circle is proportional to the percentage of patients referring to the group, cluster, or theme

**Fig.2:**
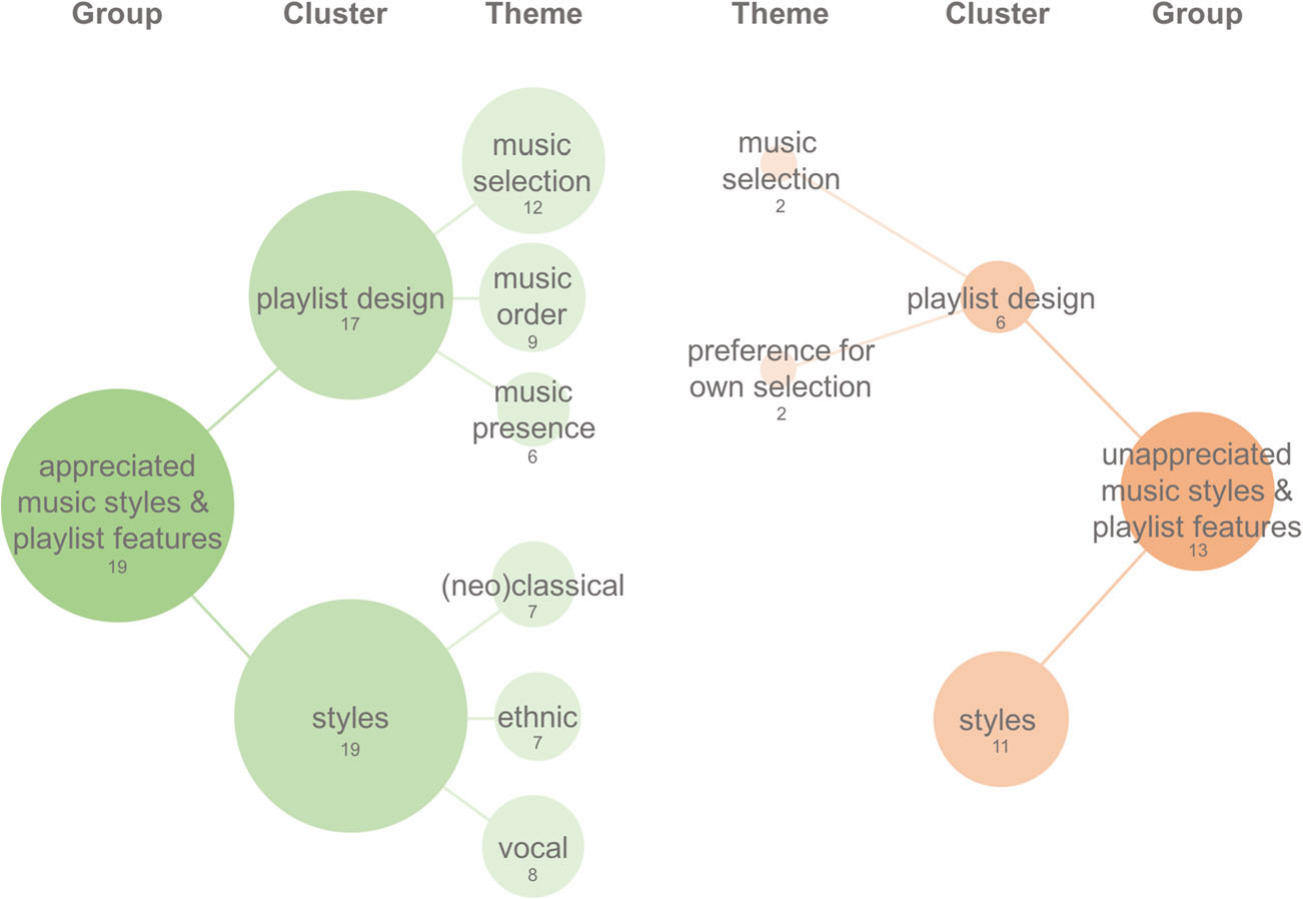
Appreciated and unappreciated music styles and playlist features. Appreciated music styles and features are displayed on the left, in green, and un-appreciated influences are displayed on the right, in red. The figure only displays cluster present in more than 30% of all participants and per cluster the themes that were present in more than 30% of the cluster. The numbers below the group-, cluster-, or theme-name refers to the total number of patients that referred to this. The size of the circle is proportional to the percentage of patients referring to the group, cluster, or theme

The 19 interview transcriptions were on average 1048 words long each (standard error = 133). Coding analyses identified a total of four separate groups, each including different clusters with related themes. These four different groups were (1) “welcome influences,” including all influences of music on subjective experience that were described as welcome, wanted, accepted, or appreciated (see Fig. 1, identified in 18 out of 19 patients, i.e. 95% of total); (2) “unwelcome influences,” including all experienced influences of music that were described as unwelcome, unwanted, rejected, or unappreciated (see Fig. 1, identified in ten out of 19 patients, i.e. 53% of total); (3) “appreciated music styles and playlist features,” including all themes related to the liking and appreciating of music genres, styles, and playlist design (see Fig. 2, identified in all 19 patients, i.e. 100% of total); and (4) “unappreciated music styles and playlist design,” including all themes related to the disliking and not appreciating of music genres, styles, and playlist design (see Fig. 2, identified in 11 out of 19 patients, i.e. 58% of total). Here, the term “music styles” refers broadly to the instrumentation, compositional, genre, and acoustic features of the music. The term “playlist design” refers to all aspects related to the selection and structuring of the music into the full music playlist.

The figures displaying the four groups (Figs. 1 and 2) include the clusters present in more than 30% of the respective groupand the themes present in more than 30% of the respective cluster. This threshold was chosen for display purposes and emphasisesthe most dominant themes. However, all themes are discussed, and all associated patient quotes are presented in separate tables in Supplementary materials (Tables 1–11). It is important to emphasise that the identification of a theme in a patient’s experience, and subsequently the including of that theme in counting its presence in the total population, does not enable to make any statements on the duration that this theme was present in the patient’s total experience. For example, one patient may have experienced a sense of irritation in response to one particular song, and therefore the theme “irritation” under the cluster “intensification” in the group unwelcomed influences is present. But this does not imply that the patient experienced persistent feelings of irritation during his or her experience: it may simply refer to one short but memorable moment. In addition, the measure also only allows the capturing of spontaneous mentioning and elaborations on the subjective experience of the music in response to the open questions, as opposed to the questions targeting (and biasing) specific facets of the experience. The only bias present within the interview that is important to acknowledge was the inquiry of both “positive” and “negative” influences of the music, leading to the subsequent “welcome” and “unwelcome” groups.

### Welcome influences: intensification

The most prominent cluster in the group welcome influences, including 17 out of 19 patients (89% of total), refers to themes that describe an intensification of the subjective experience by the music. Within this cluster, themes that describe an “intensification of emotion” were identified in 15 out of 17 (82% of cluster), including descriptions of music enhancing or changing emotions. Importantly, the emotion-evoking effects that were welcomed showed diverse emotional valence and included descriptions of the music facilitating “happiness” or strong “ecstatic” experiences, as well experiences of the music intensifying “tearfulness.”

Themes describing an “intensification of imagination” were identified in nine out of 17 (53% of cluster). This included statements of the music-evoking vivid and complex mental imagery and of the concrete imagery relating to specific characteristics of the music, such as ethnic “Indian” style of the music being associated with “seeing an Indian temple.”Eight out of 17 patients (47% of cluster) mentioned a “general intensification” effect of the music, without specifically referring to this being an intensification of emotionality, imagery, or others. Other themes, present below 30% in the cluster intensification, include effects of music on “personal thoughts or memories” (2/17, 12% of cluster), music facilitating a “sense of transcendence” (2/17, 12% of cluster), and music enhancing “ego dissolution” (2/17, 12% of cluster) (Fig. 1). See Table 1 (Supplementary materials) for a listing of all themes present in the cluster intensification.

### Welcome influences of the music: guidance

The second most prominent cluster of welcome influences includes themes that depict the music as a source of “guidance.” This cluster was mentioned by 15 out of 19 patients (79% of total). Within this cluster, statements that the music provided a “sense of being on a journey” were identified in 11 out of 15 (73% of cluster). This included descriptions of the music being experienced as a “vehicle” that “transports” or “carries” the listener forward, providing a sensation of “travelling” to different psychological “places.”

Themes describing the music as a source for psychological “support” were identified in 11 out of 15 (73% of cluster). This includes various statements of the music providing a sense of “grounding,” “help,” and “reassurance.”Descriptions of the music being in tune with, or in resonance with the person’s intrinsic emotional state, were identified in six out of 15 (40% of cluster). Rather than describing the music as evoking emotion, this theme is defined by statements of the music being experienced as “fitting,” “following,” or “matching” present emotional states.

Finally, five out of 15 of patients (33% of cluster) referred to the music as providing a “sense of continuity and direction,” this included statements of music providing a sense of connection between different parts in the experience, making the experience feel “driven” by the music and “flowing” into a certain direction (Fig. 1). See Table 2(Supplementary materials) for a listing of all themes present in the cluster guidance.

### Welcome influence of the music: calming

Ten out of 19 patients (53% of total) described calming effects of the music. From this cluster, nine out ten(90% of cluster) described “general calming” effects, whereas five out of ten patients (50% of cluster) described the music as providing “mental calming” effects, including sensations of peacefulness and of the music calming and “slowing the mind.” One out of ten (10% of cluster) described that the music helped them to feel more physically relaxed. Calming effects of music often referred to ambient music by Brian Eno, Harold Budd, and Stars of the lid. See Table 3 (Supplementary materials) for a listing of all themes present in the cluster “calming.”

### Welcome influences of the music: openness to music-evoked experience

Seven out of 19 patients (37% of total) made statements about their own attitude of openness towards the influences of the music and in addition, about the effects of music on their attitude of openness. From this cluster, six out of seven (86% of cluster) referred to the “importance” and the “purpose” of being open to “challenging experience” evoked by the music, and that this felt like an important part of the therapeutic process. This included statements of accepting being deeply emotionally moved by the music and the music helping to “face” or “connect with” the listener’s “unresolved” inner conflicts. Four out of seven (57% of cluster) described that some music specifically helped to enhance their attitude of openness, such as statements that “the music opened (him/her) up” or that because of the music was “well-chosen,” the listener “felt open to it all” (Fig. 1). See Table 4 (Supplementary materials) for a listing of all themes present in the cluster “openness to music-evoked experience.”

### Unwelcome influences of the music: intensification

The most prominent cluster, including five out of ten patients (50% of cluster), described music to “intensify” emotions they did not want to feel, such as increased “fearfulness,” “sadness,” or “fear.” In addition, five out of ten (50% of cluster) made statements about the music creating a sense of “discomfort,” including “unpleasant” or “uncomfortable” experiences, and four out of ten (40% of cluster) described irritation as a consequence of the music. In less than 30% of the cluster, the music was described as bringing mental imagery, thoughts or memories that were unwelcome, a sense of puzzlement, inner conflict, tension, or a “dark atmosphere.” This cluster of unwelcome intensification influences forms a contrast with the cluster of themes describing intensification as a welcomed influence (Fig. 1 and Table 1 (Supplementary materials)). See Table 5 (Supplementary materials) for a listing of all themes present in the cluster unwelcomed intensification.

### Unwelcome influences of the music: resistance to music-evoked experience

Nine out of 19 patients (47% of total) described feelings of “resistance to the music-evoked experience.” This includes statements of “not liking” or “not wanting” the subjective effects of the music. This cluster of unwelcomed influences contrasts the cluster of themes describing an openness to music-evoked experience, as a welcomed influence (see Table 4 (Supplementary materials) and Fig. 1). See Table 6 (Supplementary materials) for a full list of all themes in the cluster intensification.

### Unwelcome influences of the music: misguidance

Six out of 19 (32% of total) made statements about the music providing a sense of “misguidance”; this cluster primarily includes descriptions of the music being a “mismatch” or being incongruent with the unfolding subjective experience. This cluster, named “dissonance,” was present in four out of six (67% of cluster) and forms a contrast with the welcome influence resonance, when the music was experienced as harmonious, or a good match, with the subjective experience. Other themes of misguidance, present in less than 30%, include descriptions of the “music feeling intrusive,” the music being “unable to positively influence a challenging experience,” the music giving a “sense of being manipulated,” the music giving a “sense of unmet potential,” or the music giving a sense of “foreboding,” as if something “bad” was going to happen. This cluster of unwelcome influence contrasts the cluster of themes describing a sense of “supportive” and “helpful” guidance, as a welcome influence (see Table 2 (Supplementary materials) and Fig. 1). See Table 7 (Supplementary materials) for a full list of all themes in the cluster misguidance.

### Appreciated music styles and playlist features: music styles

All 19 patients referred to some music styles within the music playlist that they especially appreciated (Fig. 2). Most frequent were positive statements about “ethnic music,” present in eight out of 19 patients (42% of cluster), such as Indian, “Spanish,” or “African” music styles (e.g. Jon Hassel, Ry Cooder, and Ronu majumdar). Positive statements about music with human voice were mentioned by seven out of 19 patients (37% of cluster). Importantly, this refers to vocal music either without lyrics or music with lyrics in a foreign language (e.g. *The Journey* by Ludovico Enaudi and Enya’s *sumiregusa*). One other music style that was frequently appreciated by seven out of 19 (37% of cluster) was neo-classical music (e.g. Max Richter or Olafur Arnalds) or classical music (e.g. Henryk Gorecki or Arvo Part). Apart from these styles, the appreciated music styles showed a noticeable diversity. In less than 30%, positive statements were directed to “music with crescendo” (five out of 19, 26% of total), “powerful music” (four out of 19, 21% of total), and only one to two out of 19 made explicit statements about their appreciation for specific instruments, such as violin, guitar, piano, or “music with a solid drone.” See Table 8 (Supplementary materials) for a full listing of all themes referring to music styles that were explicitly appreciated.

### Appreciated music styles and playlist features: playlist design

Seventeen out of 19 patients (89% of total) made statements reflecting appreciation for the design of the playlist (Fig. 2). Most prominent were positive descriptions of the “music selection,” described by 12 out of 17 patients (71% of cluster), including descriptions of the music “working well” or being “well-selected.” Secondly, nine out of 17 patients (53% of cluster) provided positive descriptions on the way the music was structured into the full playlist. This theme, named “music order,” is defined by statements of the “structure” and the “ordering” of the music playlist, “aligned” well with the drug effects. The third most prominent theme, present in six out of 17 (35%), corresponds to the “music presence,” meaning the mere presence of the music itself. This includes descriptions from the music being present as helpful, to statements that it could not be imaginable doing the sessions without it and that the music presence felt “necessary.” Finally, other themes include appreciation for “calming music” to be played mainly during onset, ascent, and return phases, whereas more emotive music (i.e. “sentimental” or “cinematic” music) to be better reserved for late in the ascent phase and during peak phase. See Table 9 (Supplementary materials) for a full listing of all themes describing playlist design features that were appreciated.

### Unappreciated music styles and playlist features: music styles

Eleven out of 19 patients (58% of total) referred to musical styles that were not appreciated. These responses reflected different degrees of the individual’s disliking of the music and were highly diverse, making no theme present in more than 30% of this cluster (Fig. 2). Some examples of themes in this cluster refer to “music with lyrics,” “vocal music,” “piano music,” “classical or neo-classical music,” and “cheesy music.”Often, vocal music and cheesy music referred to one particular song played during the final return phase by Buffy Saint Mary, *up where we belong.* See Table 10 (Supplementary materials) for a list of all themes present in the cluster of un-appreciated music styles.

### Unappreciated music styles and playlist features: playlist design

Six out of 19 patients (32% of total) referred to aspects of the playlist design that were not appreciated. In 2 out of 6 (33% of cluster), a clear disliking of the music selection was present, and a preference for “own music selection” was expressed (Fig. 2). See Table 11 for a complete list of all themes present in the cluster of un-appreciated playlist design features.

### Predictors in music experience for psilocybin experience and therapy outcomes

**Fig.3:**
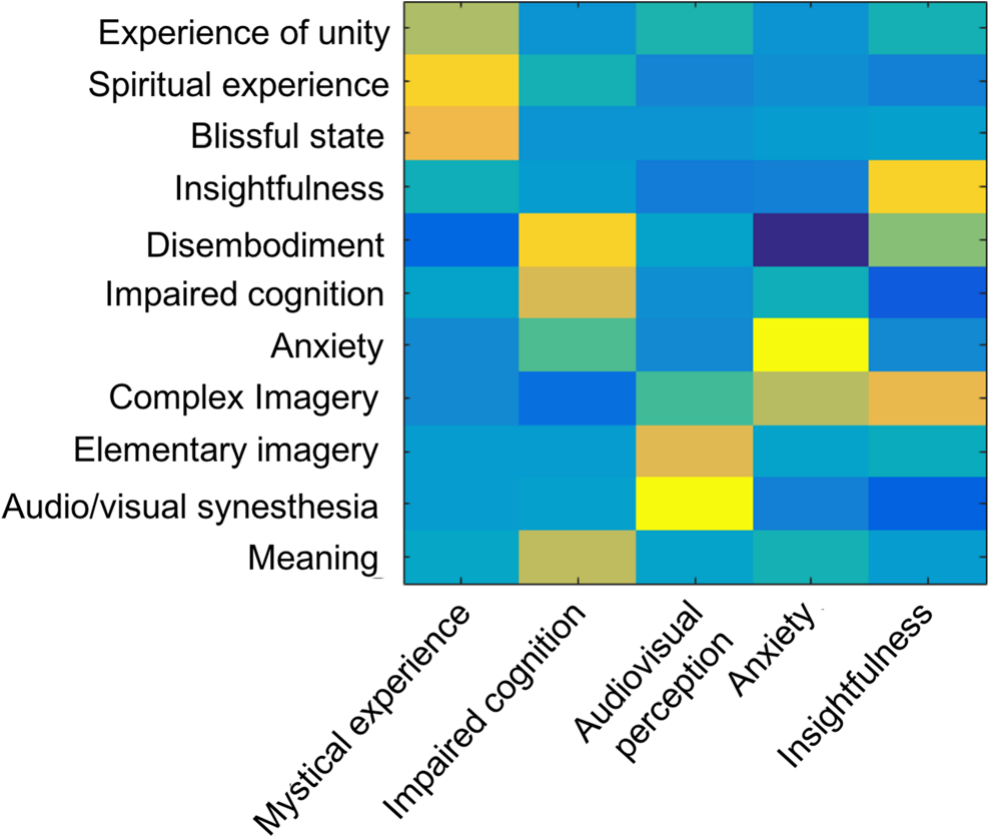
Principle component analysis (PCA) of variables from the 11D-ASC. Loadings of the 11 dimensions of the ASC (*y*-axis), on the first five PCs obtained from PCA followed by varimax rotation explained more than 95% of the variance. The *x*-axis shows the ordering of principal components, with the components ordered by explained variance (from left to right). The colour bar corresponds to the strength of the loading for each acoustic feature for that components: warm colours indicatea positive loading and cold colours a negative loading

PCA reduced the dimensions of the 11-ASC to five factors, explaining more than 95% of total variance. These PCs are (1) “mystical experience” (loadings from “experiences of unity,” “spiritual experience,” and “blissful state”), (2) “impaired cognition” (loadings from “disembodiment,”impaired cognition, and “new meanings”), (3) “audiovisual perception” (loadings from “audio/visual synaesthesia” and “elementary imagery”), (4)” anxiety” (primarily loaded by anxiety), and (5) “insightfulness” (loadings from insightfulness and “complex imagery”) (see Fig. 3). Subsequently, music experience (liking, resonance, and openness) and drug intensity scores were correlated with these five factors and ratings for reductions in depression (1 week after psilocybin, defined by % reduction in QIDS score).

**Fig.4:**
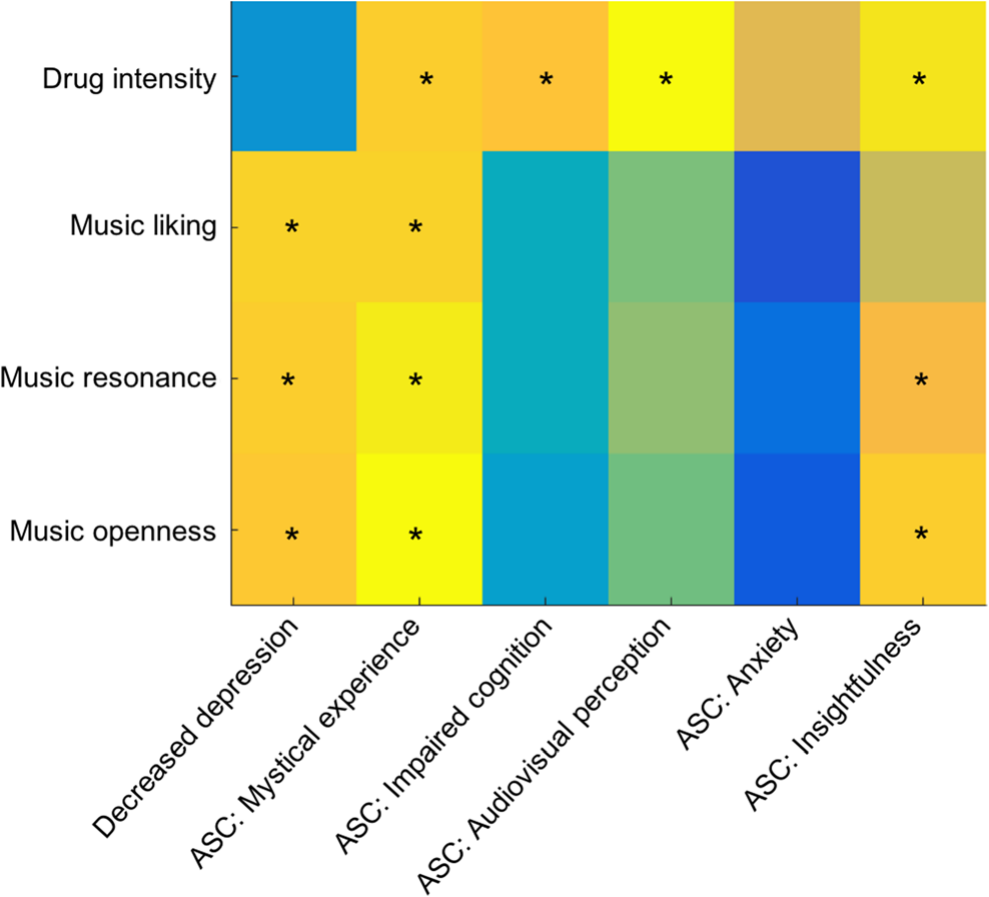
Correlations between music experience and therapy experience and outcomes. Outcomes of Pearson correlation tests of drug intensity ratings and music experience variables (on *y*-axis), with decreased depression (1 week after psilocybin) and the acute psilocybin experience (five PCs from ASC) (on *x*-axis). * = *p* < 0.05 and ** = *p* < 0.001, after FDR correction for multiple comparisons

Reductions in depression 1 week after psilocybin were significantly predicted by the musicexperience variables,liking (*r* = 0.60, *p* = .006), resonance (*r* = 0.59, *p* = .008), and openness (*r* = 0.57, *p* = .001), but not by drug intensity (*r* = 0.004, *p* = 0.98). Mystical experience during the psilocybin sessions was significantly predicted by music variables, liking (*r* = 0.61, *p* = .006), resonance (*r* = 0.67, *p* = .002), openness (*r* = 0.70, *p* = .0008), and by drug intensity (*r* = 0.58, *p* = 0.009). Insightfulness was predicted by music variables resonance (*r* = 0.53, *p* = .016) and openness (*r* = 0.59, *p* = .007), as well as by drug intensity (*r* = 0.65, *p* = 0.002), but not by music liking (*r* = 0.44, *p* = .06). Impaired cognition (*r* = 0.55,*p* = 0.01) and audiovisual perception changes (*r* = 0.71, *p* = 0.0006) were only predicted by drug intensity and not by any of the music variables. Anxiety was not predicted by any of the variables. All reported significant *p*values refer to FDR-adjusted threshold for significance of 0.016. See Fig. 4.

### Inter-rating reliability and discriminative validity of musicexperience variables

Pearson correlation tests between the scores of all researchers (*n* = 4), who rated the three musicexperience variables (liking, resonance, and openness), demonstrated good inter-rater reliability (average *r* = 0.6 ± 0.1, from total of 18 correlations). Pearson correlation tests between the three music experience variables showed significant correlations (*r* = 0.9, *r* = 0.96, and *r* = 0.91). Drug intensity did not correlate with any of the music experience variables.

### Discussion

Via an analysis of patient interviews, this study identified a number of ways in which music influenced the subjective experiences of patients receiving psilocybin with psychological support for treatment-resistant depression. The most frequently reported themes relate to an intensification of emotions and mental imagery by music under psilocybin, complementing previous studies that demonstrated modulatory effects of LSD on music-evoked emotion (Kaelen et al. 2015, 2017) and music-evoked mental imagery (Kaelen et al. 2016) in healthy volunteers. By focussing on the phenomenology of the acute experience, the present study provided new insights into the role and importance of music in the context of psychedelic therapy. For example, the music appeared to be a significant source of guidance, creating a sense of grounding, as well as a sense of carrying the listener into different psychological places. Specific examples of this can be found in the following two excerpts:

The sad songs would bring painful memories on, more happy songs would make me think of a really good period in my life. Every new song could bring a different image. (#4)

I feel the music in large part drove a lot of the experience. Under the influence of psilocybin, the music absolutely takes over. Normally when I hear a piece of sad music, or happy music I respond through choice… but under psilocybin I felt almost that I had no choice but to go with the music. […] I did feel I was being held. And it did feel like the music opened [me] up to grief, and I just was very happy for that to happen. It wasn’t particularly pleasant in any way, but extraordinarily powerful. It took my thinking and my experience to uncomfortable places, but I was kind of reassured in the experience. There was something there that meant “*I’m going to take you on a ride here, but I promise I won’t abandon you. It’s just going to be tough, and you know, you’re going through the grinder here, but you won’t be left in pieces*.” That seemed to be… what the music was saying to me. (#14).

In contrast to the sense of guidance by the music were descriptions of the music providing a sense of misguidance. In these situations, the music was most often described as being dissonant with the patient’s emotions and thoughts. One example of the experience of misguidance and dissonance can be found in the following excerpt:

The light music at one point took me to a place where I thought I was safe, and it became unsafe, and the music was playing a trick with me, you know, sort of giving me a false sense of security. I can remember thinking “*this is beautiful music, why am I going to this dark place?*” It didn’t line up with what had gone on before. I just felt as I was being manipulated, being duped almost. The music lured me to this beautiful place, and then things started to become dark even with this beautiful music still playing. (#16)

One important observation is that effects of the music that were welcomed, included emotions such as increased grieving or tearfulness, and that an attitude of openness towards negative music-evoked emotions was frequently described as helpful in bringing to expression inner psychological conflicts that might then be resolved (Watts et al. 2017). These experiences were grouped under the theme “openness to challenging experience feels therapeutic” and show similarities with recent qualitative research showing perceived therapeutic meaning in transient psychological struggle during psychedelic therapy (Belser et al. 2017; Swift et al. 2017). One example of this attitude of openness towards the music can be found in the following excerpt:

I can even view the negative moments as positive in a way because they served a purpose. The purpose was to sort of let me face the darkness, and my demons, I guess. It was beautiful at times, but also… yeah, the darker moments really helped to reflect on and connect with your unresolved shadows. (#19)

Contrasting such an attitude of openness to challenging experience is an attitude of resistance to the intensification effects of the music. This experience was characterised by not wanting the music or its effects and was named “resistance to intensification.” An example of this can be found in the following excerpt:

I worried that I let [the music] shape this sort of melancholy. There was resistance, massively, to everything, every sort of sensory input, I had a fearful response. I was afraid to open my eyes, I was afraid to do anything, I was afraid that this sort of music was the last thing I’d ever hear. (#5)

### Music styles and playlist design

The study also shed light on how different musical styles and the design of the music playlist were experienced. The choice of the music and the design of the music playlist were overall well-appreciated, with the most frequently appreciated musical genres being ethnic-, vocal-, and (neo-) classical music. Appreciation was also expressed for the design of the playlist, in particular for the calming (ambient) music, which was particularly present during the early (pre-onset and early ascent) and the final (return) phases, and at periods during peak, while more emotionally evocative music being reserved for the peak phase. This indirectly supports the therapists’ views that that an optimal playlist design is characterised by a music genre selection that is structured to match the different phases of drug experience (Barrett et al. 2017; Bonny and Pahnke 1972; Grof 1980; Richards 2015).

Strong disliking of the music selection was rare, but when this did occur it proved insightful about the possible functions of music selection: Typically, disliking of the music seemed to be associated with either a “diminishment” of psilocybin’s subjective effects, accompanied by unpleasant feelings (such as discomfort and irritation), and with an attitude of resistance, characterised by an attempt to psychologically reject and distance oneself from the music, such as detailed in the following excerpt:

The music blocked my experience and feelings. A sense of irritation, frustration, and sense of lowering mood. The majority of the songs were not my kind of music, I can’t sit with that music … I have to leave the room. I was sort of feeling bad, because I wanted to work with it. I sensed the potential for a really profound experience. I couldn’t meet that potential with music that I felt was quite mediocre. To me it didn’t feel real, so I felt quite torn. (#6)

### Music experience predicts experience and therapy outcomes

As outlined above, notable polarities were observed in the music experience, such as the music being either liked or disliked, the music being either resonant or dissonant with the patient’s experience, and the patient being either open or resistant to the influence of the music. These variables (liking, resonance, and openness) positively predicted the extent to which patients reported having mystical experiences (a factor defined as the experience of unity, blissful emotionality, and spirituality). In addition, resonance and openness, but not liking, predicted the extent to which people reported insightfulness (a factor defined by having inventive ideas, feelings of profoundness, insights, and the experience of vivid personal memories or mental images). Drug intensity, on the other hand, also correlated with other aspects of the psilocybinexperience, such as impaired cognition and audio-visual perception changes. It must be noted that liking, resonance, and openness were highly correlated and thus likely represent one construct. The absence of a significant correlation between music liking and reported insightfulness may therefore be due to a lack of statistical power.

The selective association of the music experience with mystical experience and insightfulness, and not with other subjective experiences, supports the original motivations to include music in psychedelictherapy, i.e. to promote the occurrence of therapeutically meaningful experiences. Modern studies have confirmed that psilocybin can reliably facilitate mystical experiences (Griffiths et al. 2011, 2016), and these experiences have been associated with sustained positive changes in behaviour and personality (MacLean et al. 2011) and with positive therapy outcomes (Garcia-Romeu et al. 2014; Griffiths et al. 2016; Roseman et al. 2017; Ross et al. 2016). Although these studies incorporated music-listening in combination with psilocybin, this study is the first to demonstrate that the music experience during these sessions relates to the occurrence of mystical experiences. A positive relationship was also found between the music experience and reductions in depression 1 week after the psilocybin experience. Importantly, reductions in depression were not related to the intensity of the drug effects. This finding indicates that it is not merely the drug effect in isolation, but an interaction between the drug and the music on subjective experience that promotes positive therapeutic outcomes.

### Possible therapeutic mechanisms of music in psychedelic therapy

A principal effect of psychedelics is that they temporarily dysregulate brain mechanisms that normally regulate emotion(Carhart-Harris et al. 2012a, 2016b; Muthukumaraswamy et al. 2013; Tagliazucchi et al. 2016), and this could underlie the enhanced emotional responsiveness to emotionally evocative stimuli reported here as elsewhere (Carhart-Harris et al. 2012b; Kaelen et al. 2015, 2017; Quednow et al. 2012; Vollenweider et al. 2007). The notion that accepting and moving through challenging emotions are important for psychotherapeutic change is central to many psychotherapeutic models (Greenberg and Pascual-Leone 2006), has empirical support (Whelton 2004), and been noted by other psychedelic therapy studies (Belser et al. 2017; Swift et al. 2017; Watts et al. 2017). In psychedelic therapy, the function of psychedelics may be to ease the relinquishment of psychological control (i.e. ego dissolution and enhanced suggestibility (Carhart-Harris et al. 2014)), thereby allowing a fuller and freer (i.e. less inhibited) expression of emotionality. The enhanced receptivity to music, in turn, may play the important function of activating emotionality, thoughts, and memories that are most personally salient. Thereby, music can guide the patient’s experience into directions that are most therapeutically significant. One key difference between psychedelic therapy and other forms of psychotherapy (and conventional pharmacotherapy) may be the capacity of psychedelics and music to rapidly facilitate deeply felt and personally meaningful emotionality (Carhart-Harris et al. 2016a; Gasser et al. 2014; Griffiths et al. 2016; Grob et al. 2011; Johnson et al. 2014; Ross et al. 2016).

It is worth considering that these findings show a remarkable congruency with the theoretical frameworks and patient experiences of “introspective” forms of music therapy, where music is utilised as the means to provide an experience that is thought to help the listener examine and change his/her relationship with themselves (Abbott 2005; Albornoz 2013; Summer 1992, 2011). This includes the use of music to evoke intense emotional experiences (Albornoz 2013), as well as a way to provide a “holding environment,”which feels “safe and secure” to express and experience new aspects of oneself (Carroll 2011; Schulberg 1999). Therapeutic effects of music are widely reported in literature and utilised across different health care disciplines (Finch and Moscovitch 2016; Mondanaro et al. 2017; Pavlov et al. 2017). The present findings therefore engender the view that psychedelic therapy utilises therapeutic effects of music that are enhanced via an interaction between the drug and the music.

### Implications for the use of music in psychedelic therapy

Due to the prominence of music-listening in psychedelic therapy, increasing the knowledge of the appropriate therapeutic use of music in psychedelic therapy is important. This becomes particularly critical when psychedelic therapy is implemented on increasingly larger scales. The therapeutic influence of music has been referred to as being of “*profound significance*”(Bonny and Pahnke 1972), and several authors emphasised the care needed in selecting appropriate music, playing this music at the right circumstances, and within a personalised patient-centred format (Grof 1980; Hoffer 1965). The present study provides support for these views, by showing that when the music was experienced as dissonant with the unfolding experience, disliked, and rejected (resistance), therapeutic outcomes suffered. In contrast, when the music was in resonance with the patient’s experience, liked, and accepted (openness), therapeutic outcomes were most positive.

These music experience variables in this study (resonance, liking, and openness) correlated with each other, suggesting that they represent a single construct within the music experience that is associated with positive therapy outcomes. Liking of music is usually characterised as a mixture of genre appreciation and aesthetic judgements (Juslin 2013; Juslin and Västfjäll 2008; Juslin et al. 2016; North and Hargreaves 1997), and music liking may represent a basic pre-requisite for music to evoke personally meaningful emotionality. In addition, some music styles and acoustic properties may be more suitable for the conscious states induced by psychedelics than others. The patient’s attitude, in turn, appears to require a sufficient degree of openness to the music-evoked experience, and this may imply not only a state of surrender but also a pro-active and curious engagement with the therapeutic content that emerges.

This hypothetical framework holds that an optimal music experience (style liking, music’s resonance, and openness to music) creates an optimal climate for the expression of meaningful therapeutic content, characterised by the sensation of being on a personal journey, with a spontaneous and often intense emergence of personally meaningful imagery, thoughts, and emotionality. This optimal music experience construct may be a critical pre-requisite, and when it is not met adequately, is likely to result in the patient to distance from the music experience (resistance), characterised by feelings of discomfort, and a diminishment of personally meaningful imagery, thoughts, and emotionality (i.e. the absence of the sense of being on a journey). Given the patient’s experience is highly individual and dynamic, this finding suggests that the adaptation of the music during psychedelic therapy sessions may be critical at times, in order to provide adequate therapeutic support conditions, or prevent possible counter-therapeutic experiences: an idea that was often emphasised by early pioneers of psychedelic therapy (Bonny and Pahnke 1972; Grof 1980; Hoffer 1965).

In this framework, the experience of resistance and dislike by the listener may be regarded as an important indicator for the therapist of music’s failure to act therapeutically, and the type of intervention needed to restore music’s therapeutic function may be determined by one central question the therapists may need to clarify, i.e. *what is the source of the resistance or dislike*? The therapists bear a responsibility to ensure the music styles are sufficiently liked, via thoughtful music selection, and that resonance is maximised by providing an attunement of the music to the patient’s personal and dynamically unfolding experience, via thoughtful playlistdesign and adaptation of the music when needed. However, in addition, it may occur that the music-evoked experience is rich with therapeutically meaningful content, yet the experience may be emotionally challenging, resulting in similar expressions of resistance. In these scenarios, the therapists may instead need to provide adequate therapeutic support for the patient to feel safe and motivated to engage in exploring and expressing the present challenging feeling states, of which the meanings may not always be immediately clear.

### Limitations and future directions

This study has a number of limitations. First of all, the data was acquired without a placebocondition, making causal inferences about the nature of the effects problematic. Secondly, the main body of data used for this study was qualitative in nature. Therefore, the experiment did not allow studying the magnitude of the observed themes in the music experience. It should therefore be emphasised that the primary objective of this study was to provide a patient perspective on the influence of music. We hope that this work inspires new hypotheses for future studies, and that it assists therapists and researchers in their use of music in psychedelic therapy. Examples of future directions include testing whether maximization of resonance could improve therapy outcomes, and whether the variables liking, resonance, and openness represent one single factor or separate factors when larger sample sizes and more precise measurements are employed.

A significant body of empirical work is required to advance the therapeutic use of music in psychedelic therapy. One important focus of such work will be the establishing of baseline measures that can reliably predict individual music experiences during psychedelic therapy sessions. Such predictive measures can range from personality traits (e.g. openness to experience, absorption, or suggestibility) to measures of personal music preferences. Furthermore, research that focuses on identifying reliable indicators of positive (welcome/supportive) and negative (unwelcome/unsupportive) influences of music on the therapeutic processes during psychedelic therapy sessions may help therapists adapt music to individual patients.

## Conclusions

In patients with treatment-resistant depression treated with psilocybin, music was described as having a substantial influence on their therapeutic experience, and selective correlations between the musicexperience and the occurrence of mystical experiences and insightfulness during sessions support this. Patients’ experience of the music, but not drug intensity, was predictive of reductions in depression 1 week later, suggesting that music plays a central mediating role in psychedelic therapy.These findings motivate greater appreciation of music as a key variable in psychedelic therapy and highlight the need for further research to better understand how music interacts with certain personality traits and psychological states to influence the acute experience and longer-term outcomes of psychedelic therapy.

## Electronic supplementary material

ESM 1 (DOCX 72 kb)

## Footnotes

1 The term mystical experience is derived from writings on religious mysticism and should not be confused with the terms “mystery” or “mysteriousness”: Mystical experiences refer in essence to a direct felt experience of “union” and a sense of transcending one’s usual sense of self (Stace 1960). Although we initially considered using the more secular term “peak experience”coined by the psychologist Abraham Maslow (Maslow 1964, 1971), which essentially refers to a comparable and if not identical phenomenological state, we have decided after discussion with our reviewers to employ the term mystical experience. This term is currently the most used in the academic field (Garcia-Romeu et al. 2014; Ross et al. 2016; Griffiths et al. 2016), and we do not wish to cause any unnecessary confusion over used terminologies. Mystical experiences are by definition not specific to any religion and can occur spontaneously, such as when being deeply moved by a work of art, encounters with nature, during sex, or a moment of creative inspiration, but are frequently reported with psychedelics. In the present study, although we did not employ the Mystical Experience Questionnaire (Maclean et al. 2012), our use of the term was defined by the items from the Altered State of Consciousness Scale(ASC), “experiences of unity,” “spiritual experience,” and “blissful state” (Dittrich 1998; Studerus et al. 2010), that loaded on one primary factor using principle component analysis.
