# Correction to: The hidden therapist: evidence for a central role of music in psychedelic therapy

**DOI:** 10.1007/s00213-018-4886-8

**Published:** 2018-03-26

**Authors:** Mendel Kaelen, Bruna Giribaldi, Jordan Raine, Lisa Evans, Christopher Timmermann, Natalie Rodriguez, Leor Roseman, Amanda Feilding, David Nutt, Robin Carhart-Harris

**Affiliations:** 1 Psychedelic Research Group, Department of Medicine, Imperial College London, W12 0NN, London, UK; 2 School of Psychology, Sussex University, Brighton, BN1 9RH UK; 3 Computational, Cognitive and Clinical Neuroscience Laboratory (C3NL), Department of Medicine, Imperial College London, W12 0NN, London, UK; 4 The Beckley Foundation, Oxford, OX3 9SY UK

**Correction to: Psychopharmacology**

**https://doi.org/10.1007/s00213-017-4820-5**

The article The hidden therapist: evidence for a central role of music in psychedelic therapy, written by Mendel Kaelen, Bruna Giribaldi, Jordan Raine, Lisa Evans, Christopher Timmerman, Natalie Rodriguez, Leor Roseman, Amanda Feilding, David Nutt, Robin Carhart-Harris, was originally published electronically on the publisher’s internet portal (currently SpringerLink) on 2 February 2018 without open access.

With the author(s)’ decision to opt for Open Choice the copyright of the article changed on 19 March 2018 to © The Author(s) 2018 and the article is forthwith distributed under the terms of the Creative Commons Attribution 4.0 International License (http://creativecommons.org/licenses/by/4.0/), which permits use, duplication, adaptation, distribution and reproduction in any medium or format, as long as you give appropriate credit to the original author(s) and the source, provide a link to the Creative Commons license and indicate if changes were made.

Also, Christopher Timmerman should have been Christopher Timmermann

