# Supplementary material for: The hidden therapist: evidence for a central role of music in psychedelic therapy
Source: Psychopharmacology (Berl). 2018 Feb 2;235(2):505–19. doi: 10.1007/s00213-017-4820-5 (PMC5893695; doi:10.1007/s00213-017-4820-5)
Supplement: Supplementary file 1 — (DOCX 72 kb) [file 213_2017_4820_MOESM1_ESM.docx]

**Supplementary materials**

**Table 1 | Welcomed influences: Intensification*.*** A list of all the themes and respective quotes by patients, that were present in the cluster “intensification”

| **#** | **Quote** | **Theme** |
| --- | --- | --- |
| 1 | “The level of intensity of the music made things in my mind seem more intense.” | General intensification |
| 3 | “[The music] can amplify the emotions, either negatively or positively.” | Emotionality |
|  | “Make you feel happy when there’s a good song on.” | Emotionality (happiness) |
|  | “it was all good […] all in the same area where it can get people meditating, or releasing, or just chilling out to it.” | Emotionality |
|  | “Your emotion goes with that [music] and the happy feeling, and its like all connected.” | Emotionality (happiness) |
| 4 | “[The music] made me even more emotional.” | Emotionality |
|  | “The sad songs would bring painful memories on, more happy songs would make me think of a really good period in my life.” | Personal thoughts or memories |
|  | “I didn’t like the emotional music the first experience cause I didn’t want to cry, but actually, you know, the second time I sort of enjoyed crying to this music.” | Emotionality (tearfulness) |
|  | “[The soothing music] brought up nice images, like I imagined I was an unborn child and I felt very safe. Every new song could bring a different image, and I couldn’t finish the previous one that I was thinking about. It was interesting.” | Imagination |
| 5 | “[The music] made me see what I considered ephemeral things, like a sense of a mother and a sense of a child. And their behaviour was definitely informed by the music.” | Imagination |
|  | “[The music] caused different feelings.” | Emotionality |
|  | “[The music] caused the different imagery.” | Imagination |
| 6(2) | "There were moments when I felt just this burst of joy." | Emotionality (happiness) |
|  | "And I connected [the music] very strongly thinking about Rembrandt who I love." | Personal thoughts or memories |
|  | "So I just felt overwhelmed with joy and gratitude to have that [music] in my life." | Emotionality (gratitude) |
|  | "There was one piece that I knew very well and it reminded me of somebody." | Personal thoughts or memories |
| 7 | “Some bits of it were otherworldly.” | Imagination |
|  | “I could almost picture myself there.” | Imagination |
|  | “[The music] seemed to heighten the experience.” | General intensification |
|  | “[The music] stirred something.” | Emotionality |
|  | “[The music] seemed to fit the space that I was inhabiting. I felt a dissolution, where you sort of dissolve. And I felt the music after the quiet ambient music, in the beginning, emphasized that beautifully. And it definitely enhanced it.” | General intensification, Ego-dissolution |
| 9 | “There's one track [Greg Haines - Azure] that gets really really really intense.” | General intensification |
|  | “[Greg Haines - Azure] I felt like I was sort of the highest I could get, it was like the absolute, the top of everything.” | Sense of transcendence |
|  | “[Greg Haines - Azure] really really beautiful, overwhelming, but really peaceful as well.” | Emotionality |
| 10 | “[The music] helped me to emotionally connect.” | Emotionality |
|  | “I wouldn’t say that I felt sad, but, I obviously cried, but I don’t know why I did (half-laugh)- but I wasn’t sad.” | Emotionality (tearfulness) |
|  | “if I’d listened to anything else then I probably would have had a different experience [emotionally].” | Emotionality |
|  | “At the beginning, as the effects starts to, you know, creep up on me, it was quite “Chinese-ey”? And (giggles) the bit where I saw the psychedelic Chinese dragon.” | Imagination |
|  | “I was tearful at some points.” | Emotionality (tearfulness) |
| 11 | “In terms of movement, of the patterns going along with the music.” | Imagination |
|  | “Indian music, where I was seeing an Indian temple.” | Imagination |
|  | “Setting the place, an Inca princesses singing and seeing the terraced Inca-type setting.” | Imagination |
|  | “The music was influencing the movements and influencing the setting.” | Imagination |
|  | “During that same Indian piece, that’s when I was dancing for Shiva and then I was Shiva.” | Imagination |
|  | “[The music] influenced this sensory perception.” | Imagination |
|  | “Saying that it made me sad or it made me happy, those words are actually not irrelevant, a little too mild almost to explain what it was.” | Emotionality |
|  | “I felt ecstasy, “ek-stasis”. It was hard to separate out what’s happening here, from what’s happening here, from what’s happening here.” | Emotionality (ecstasy) |
| 12 | “[The music] helped with the visual stuff and the mood.” | Imagination, Emotionality |
|  | “the visuals … [The music] kind of opens up your imagination in a way, your imagination is much easier to picture. One of the songs created imagery of a "wooden bannister" that had a shape, it had a texture and a material, with some water and marbles with rainbows in it. I could picture it really clearly.” | Imagination |
|  | “[the music and the experience] was a symbiotic kind of thing.” | General intensification |
|  | “That piece of music was probably the peak of positivity, it was almost like a subdued kind of ecstasy, you know, it was so amazing. It was such a beautiful feeling.” | Emotionality (ecstasy) |
|  | “[Experiencing a challenging influence of the music] wasn’t a bad thing you know, it opened you up.” | Openness |
|  | “The music is kind of changing your state of mind almost, or putting you on a different resonance.” | General intensification |
| 13 | “It feels like your mood, or the the intensity of the drug, it feels like it goes with the music.” | Emotionality, General intensification |
| 14 | “Under the influence of psilocybin,[the music] absolutely takes over.” | General intensification |
|  | “Normally when I hear a piece of sad music, or happy music, I respond through choice, but under psilocybin I felt almost that I had no choice but to go with the music.” | Emotionality, Ego-dissolution |
|  | “And I did feel like [the music] opened up to grief, and I just was very happy for that to happen.” | Emotionality (tearfulness) |
|  | “Primitive music felt like it was taking me under, I had no choice.” | Ego-dissolution |
|  | “The patterns of the sound were doing something to me visually, and I responded emotionally to the visuals, and the visuals were directly related to the sound.” | Emotionality, Imagination |
| 15 | “It’s so beautiful it just made me cry.” | Emotionality (tearfulness) |
| 16 | “It changed the sort of imagery in my head, it didn’t make it any better or worse, but it had a definite impact” | Imagination |
|  | “It felt like everything added towards the experience rather than took away” | General intensification |
| 17 | “The music evoked a lot of emotions.” | Emotionality |
|  | “I had a sort of ecstatic experience.” | Emotionality (ecstasy) |
|  | “That seemed to be the point at which I was kind of carried away, in this ecstatic rapture.” | Emotionality (ecstasy) |
|  | “Clearly the music enhanced the emotional experience.” | Emotionality |
|  | “I suppose church-type music, I think was responsible for enhancing the religious experience.” | Emotionality (spirituality) |
| 18 | “My experience seemed sometimes to coincide with the lightness of the music into the light place and the darkness of the music to the dark.” | Emotionality |
|  | “There was a specific moment when I felt as though I was being given birth to the universe, and I semi-recall there was this sort of crescendo of music at that point, that was a very strong point in the journey for me.” | Imagination, Sense of transcendence |
|  | “When I was born into the universe, you know, everything dropped away apart from these slight tethers that were holding me back, and I was into the vastness of the universe, and that could have been when the music stopped, because I remember at that particular point looking at this immense void in front of me.” | Sense of transcendence |
| 19 | “The music did affect [the experience].” | General intensification |
|  | “The music just made it more emotional.” | Emotionality |
|  | “Yeah, it definitely served the function of helping emotions flow.” | Emotionality |

**Table 2 | Welcomed influences: Guidance.** A list of all the themes and respective quotes by patients, that were present in the cluster “guidance”.

| **#** | **Quote** | **Theme** |
| --- | --- | --- |
| 1 | “I think that it was comforting, knowing that the music was there, because it was there the whole period, the music was always in the background, so in in a way it grounded me.” | Sense of support |
|  | “Having the music in the background, it somehow just was helpful.” | Sense of support |
|  | “[The music] gave me a sense of safety during the experience.” | Sense of support |
|  | “[The music] was structured in line with the way the effects of the drug were.” | Resonance |
|  | “It was there throughout, I think it was just that continuity.” | Sense of continuity and direction |
| 2 | “The music was spot on with my feeling.” | Resonance |
|  | “[The music] was following my emotion at the time.” | Resonance |
|  | “I could see that there was a great effort to put the music together in a way that it followed the experience as it was meant to be.” | Resonance |
|  | “[The music helped with the] relaxation I went through.” | Sense of support |
| 4 | “When I had this difficult moment and [I was] encouraged to focus on the music and I put the headphones on, the song that came up was very soothing and it made me calm down and get out of that.” | Sense of support |
|  | “[The soothing music] brought up nice images, like I imagined I was an unborn child and I felt very safe. Every new song could bring a different image, and I couldn’t finish the previous one that I was thinking about. It was interesting.” | Sense of support |
| 6(2) | "It all just flowed." | Sense of continuity and direction, Sense of support |
| 7 | “It began quite ambient-like, quite relaxing, which I felt was good. It calmed you in the experience.” | Sense of support |
|  | “[The music] made me feel that I was in different locations.” | Sense of being on a journey |
|  | “I could almost picture myself there.” | Sense of being on a journey |
|  | “I felt as if I was travelling.” | Sense of being on a journey |
| 9 | “And the music felt really open itself.” | Resonance |
|  | “[Greg Haines - Azure] just builds and builds. You're holding on to an extent, you just kind of go up and you're like ‘ok, where am I? Can I go any further?’” | Sense of being on a journey |
|  | “That saying, going in and through it, really helped me throughout the whole experience.” | Sense of support |
| 10 | “it was all kind of a journey.” | Sense of continuity and direction, Sense of being on a journey |
|  | “It was seamless- apart from the breaks. But after breaks they would put the headphones in and I would go straight back into that world.” | Sense of continuity and direction |
|  | “Without it I would have gone mad.” | Sense of support |
| 11 | “The most facilitative to the trip were the straight on classical pieces.” | Sense of support |
| 12 | “[The music] helped with the visual stuff and the mood.” | Sense of support |
|  | It was like a rollercoaster, you move with the music.” | Sense of being on a journey |
|  | “You were led up and then brought down and led up.” | Sense of being on a journey |
|  | “The music is kind of changing your state of mind almost, or putting you on a different resonance.” | General intensification |
| 13 | “I think the way it was guiding me. It was good.” | Sense of support |
|  | “It’s [the music] definitely necessary. It kind of cuts you off from where you are at that time.” | Sense of being on a journey |
| 14 | “I feel the music in large part drove a lot of the experience.” | Sense of continuity and direction |
|  | “I did feel as if I was being held” | Sense of support |
|  | “[The music] took my thinking and my experience to uncomfortable places, but I was kind of reassured in the experience.” | Sense of support |
|  | “There was something there that meant that, you know, ‘I’m going to take you on a ride here, but I promise I won’t abandon you. It’s just going to be tough, and you know, you’re going through the grinder here, but you won’t be left in pieces.’ That seemed to be what the music was saying to me.” | Sense of support, sense of being on a journey |
| 16 | “The music took you to the places you needed to be.” | Sense of support, sense of being on a journey |
|  | “It felt we had a profound relationship, between my heartbeat and the beat of the music.” | Resonance |
|  | “It felt like the music picked you up and carried you to the next part, and the next piece, and it was the vehicle that moved you.” | Sense of being on a journey, Sense of continuity and direction |
|  | “There was a point and I didn’t want music anymore, I didn’t want anything but I definitely think the music sort of transported there to where you should be” | Sense of being transported |
|  | “It felt like it all fitted the experience” | Resonance |
| 17 | “That seemed to be the point at which I was kind of carried away, in this ecstatic rapture.” | Sense of being on a journey |
|  | “I was able to just put the headphones on and the mask on, and really enjoy it.” | Sense of support |
|  | “The music definitely assisted me.” | Sense of support |
| 18 | “The music both took me to very beautiful places and to incredibly dark places.” | Sense of being on a journey |
|  | “My experience seemed sometimes to coincide with the lightness of the music into the light place and the darkness of the music to the dark.” | Resonance |
|  | “I went to a very dark place.” | Sense of being on a journey |
|  | “It was a woman’s voice, it was a sort of operatic voice, and first it was beautiful because, you know, I was following it and it took me to a beautiful place.” | Sense of being on a journey |
| 19 | “Yeah, it definitely served the function of helping emotions flow.” | Sense of support |

**Table 3 | Welcomed influences: calming.** A list of all the themes and respective quotes by patients, that were present in the cluster “calming”.

| **#** | **Quote** | **Theme** |
| --- | --- | --- |
| 1 | “The music helped me to be relaxed.” | General calming |
|  | “My body, my breathing, was better when the medicine was wearing off, because the music was very mellow.” | Physical calming |
| 2 | “There was a part of the experience where I felt really relaxed. It was a very relaxed music and I was feeling very relaxed, so definitely there was an influence there.” | General calming |
|  | “[The music helped with the] relaxation I went through.” | General calming |
| 3 | “it was all good […] all in the same area where it can get people meditating, or releasing, or just chilling out to it.” | General calming, Mental calming |
| 4 | “When I had this difficult moment and [I was] encouraged to focus on the music and I put the headphones on, the song that came up was very soothing and it made me calm down and get out of that.” | General calming, Mental calming |
| 6(1) | "It was relief, because I hadn't liked the ones before." | General calming |
|  | "Just relief really when I just felt oh, its ok, its ok." | General calming |
| 7 | “It began quite ambient-like, quite relaxing, which I felt was good. It calmed you in the experience.” | General calming |
|  | “It did help to calm and slow the mind.” | Mental calming |
| 9 | “[Greg Haines - Azure] *really really* beautiful, overwhelming, but really peaceful as well.” | Mental calming |
| 10 | “More often than not I was sort of peaceful.” | Mental calming |
|  | “I was really calm and peaceful and relaxed.” | General calming, Mental calming |
| 13 | “And particularly once it starts to mellow out towards the end, you do feel that more relaxed.” | General calming |
| 15 | “There was a piece that was quite calming, so that was very positive.” | General calming |

**Table 4 | Welcomed influences:** **openness to music-evoked experience.** A list of all the themes and respective quotes by patients, that were present in the cluster “intensification”.

| **#** | **Quote** | | **Theme** | |
| --- | --- | --- | --- | --- |
| 3 | “That’s not what it’s about, just skipping to the next track to the good stuff. It’s there, that you don’t like it, then deal with it (chuckles), so that’s our approach.” | | Openness to challenging experience feels therapeutic | |
| 4 | “I didn’t like the emotional music the first experience because I didn’t want to cry, but actually, you know, the second time I sort of enjoyed crying to this music.” | | Openness to challenging experience feels therapeutic | |
|  | “In the first experience I was a bit annoyed that this music is too emotional, but I’m sort of thinking it was good for me, so I wouldn’t consider it as bad in the end.” | | Openness to challenging experience feels therapeutic | |
| 6(2) | "It all flowed, I opened myself to it, and it was totally positive." | | Music facilitates Openness | |
| 9 | “As far as positivity goes it was amazing. Yeah, *really, really, really* helped to open me up... [The music] really opened things up.” | | Music facilitates Openness | |
|  | “I can even view the negative moments as positive in a way because they served a purpose. The purpose was to sort of let me face the darkness, and my demons, I guess. It was beautiful at times, but also… yeah, the darker moments really helped to reflect on and connect with your demons, your unresolved shadows.” | | Openness to challenging experience feels therapeutic | |
|  | “[Henry Gorecki – Symphony of sorrowful songs] was uncomfortable, but not bad. That was necessary I feel.” | | Openness to challenging experience feels therapeutic | |
|  | “There's the odd chord change as well within other parts of the music, that affected me negatively, but that all served a purpose.” | | Openness to challenging experience feels therapeutic | |
|  | “It wasn't like having Justin Bieber for 6 hours, ‘everything's jolly and light and bright and saccharine!’” | | Openness to challenging experience feels therapeutic | |
|  | “It was such a well chosen playlist I felt that I was opened to all of it." | | Music facilitates openness | |
| 12 | “[Experiencing a challenging influence of the music] wasn’t a bad thing you know, it opened you up.” | | Openness to challenging experience feels therapeutic | |
|  |  |  | Music facilitates Openness | |
| 14 | “And I did feel like [the music] opened up to grief, and I just was very happy for that to happen.” | | Openness to challenging experience feels therapeutic | |
|  |  |  | Music facilitates Openness | |
|  | “It wasn’t particularly pleasant in any way, but extraordinarily powerful.” | | Openness to challenging experience feels therapeutic | |
| 16 | “There was a point and I didn’t want music anymore, I didn’t want anything, but I definitely think the music sort of transported there to where you should be” | Openness to challenging experience feels therapeutic | |  |

**Table 5 | Unwelcomed influences: intensification.** A list of all the themes and respective quotes by patients, that were present in the cluster “intensification”.

| # | Quote | Theme |
| --- | --- | --- |
| 3 | “It was crazy, it was like: change the song, please change the song! [Interviewer: Like a discomfort?], big time.” | Discomfort |
| 4 | “Very trippy music, making me more scared when I had this difficult phase, that I was thinking ‘ah, I’m going crazy!’” | Emotionality (fear) |
|  | “In the first experience I was a bit annoyed that this music is too emotional” | Irritation |
|  | “I didn’t like the emotional music the first experience because I didn’t want to cry.” | Emotionality (tearfulness) |
| 5 | “My response to the music was one of fear, I guess, in part, because I viewed part of the music as this really sombre, serious, negative, cynical way of thinking, like it was my funeral, or like something really profound was about to happen, or death. And it was the music that was informing that feeling.” | Emotionality (fear, sadness), Imagination, personal thoughts or memories |
|  | “In the period where it wasn’t so intense, when I cried a lot, the music elicited that sort of response.” | Emotionality (tearfulness) |
|  | “It accentuated any sense of like… emotional music, it just made me even sadder.” | Emotionality (sadness) |
| 6(1) | "A sense of irritation, frustration, and sense of lowering mood." | Irritation, Emotionality (sadness) |
|  | "It's music that I can't listen to, I find it irritating or agitating." | Irritation |
|  | "I felt quite torn." | Inner conflict |
| 7 | “Creating puzzlement rather than just accepting the music. […] ‘Why is that person singing it in that particular way?’ and I felt myself spending a lot of time thinking about that. Trying to work out why she was singing it in that particular style.” | Puzzlement |
| 9 | “[Some of the music influenced the experience by] making things seem darker.” | Dark atmosphere |
|  | “Uncomfortable chord changes.” | Discomfort |
| 13 | “With a higher tempo everything feels a bit more tense.” | Tension |
| 15 | “I wanted to explore a certain experience or vision, but the music changed that. I was experiencing these geometric shapes which were seeming to construct themselves into something that I was beginning to understand, but then the music interrupted that and changed it and I was slightly annoyed by that.” | Irritation |
|  | “The piano pieces, irritated me. It was like little needles.” | Irritation, Discomfort |
|  | “The piano playing was irritating, because I found it quite amateurish.” | Irritation |
| 17 | “There was some sort of Indian-style music, I think I found it a bit creepy at the time.” | Emotionality (fear) |
| 19 | “Made me a bit more emotional, more vulnerable.” | Emotionality |
|  | “A bit annoying” | irritation |
|  | “What I was experiencing at the time, it was you know, unpleasant” | Discomfort |
|  | “Made me feel ‘uurgh’” | Irritation |
|  | “Some of [the music] was uncomfortable.” | Discomfort |

**Table 6 | Unwelcomed influences: resistance to music-evoked experience.** A list of all the themes and respective quotes by patients, that were present in the cluster “resistance to music-evoked experience”.

| **#** | **Quote** | **Theme** |
| --- | --- | --- |
| 3 | “It was crazy, it was like: change the song, please change the song! [Interviewer: Like a discomfort?], big time.” | Music facilitates resistance |
| 4 | “I didn’t like the emotional music the first experience because I didn’t want to cry.” | Music facilitates resistance |
| 5 | “I worried that I let [the music] shape this sort of melancholy.” | Music facilitates resistance |
|  | “There was resistance, massively, to everything, every sort of sensory input I had a fearful response. I was afraid to open my eyes, I was afraid to do anything, I was afraid that this sort of music was the last thing I’d ever hear.” | Music facilitates resistance |
|  | “But I listen to a lot of similar kind of music on my own, so obviously, it does move me, whether or not I admit that, it does shape my emotions and my moods.” | Music facilitates resistance |
| 6(1) | "I was trying to suppress [the anger]." | Music facilitates resistance |
| 7 | “Creating puzzlement rather than just accepting the music. […] ‘Why is that person singing it in that particular way?’ and I felt myself spending a lot of time thinking about that. Trying to work out why she was singing it in that particular style.” | Music facilitates resistance |
| 9 | “There were times when I thought ‘I don't like the effect of this, on me.’” | Music facilitates resistance |
| 14 | “I noticed I didn’t wanna hang around for the last hour, that version of Joe Cocker, ‘Love Lift Us Up’.” | Music facilitates resistance |
|  | “That is my issue sometimes, not being willing to go with the experience [of disliked music], but there’s a lesson in there for me.” | Music facilitates resistance |
| 15 | “It was very difficult to totally let go because I thought that, I felt that if I totally let go I might not be able to bring myself back.” | Music facilitates resistance |
| 16 | “There was a point, and I didn’t want music anymore, I didn’t want anything.” | Music facilitates resistance |

**Table 7 | Unwelcomed influences: misguidance.** A list of all the themes and respective quotes by patients, that were present in the cluster “misguidance”.

| **#** | **Quote** | **Theme** |
| --- | --- | --- |
| 5 | “My response to the music was one of fear, I guess, in part, because I viewed part of the music as this really sombre, serious, negative, cynical way of thinking, like it was my funeral, or like something really profound was about to happen, or death. And it was the music that was informing that feeling.” | Sense of foreboding |
|  | “Everything was like ‘aaaaahh you’re about to die!’ (singing in dramatic voice).” | Sense of foreboding |
| 6(1) | "To me [the music] didn't feel real." | Dissonance |
|  | "I was sort of feeling bad, because I wanted to work with it." | Sense of unmet potential |
|  | "I sensed the potential for a really profound experience, but I couldn't meet [that potential] with music that I felt was quite mediocre." | Sense of unmet potential |
| 14 | I’ve heard classical music perform live and wonderfully, but I’m kind of suspicious of it now. There’s something malevolent in it, maybe not totally, but from its inspirations, because it was intended to be nationalistic and patriotic.” | Sense of being manipulated |
| 15 | “I was very aware of the influence of the music, which I found at times intrusive.” | Music feeling intrusive, Dissonance |
|  | “The music was discordant” | Dissonance |
|  | “I just found it [the piano music] grating. Intrusive even. Maybe the way it was being played.” | Music feeling intrusive |
| 18 | “I went to a very dark place with that beautiful music still playing for a while, and then it got darker. The music didn’t get dark and then I went to the dark place, I went to the dark place with the light mystical music.” | Dissonance, Music unable to positively influence challenging experience |
|  | “There was a kind of out-of-place piano, that was almost sort of ‘hunky-tunky’, but that just didn’t sort of fit really.” | Dissonance |
|  | “I think the music lured me to this beautiful place, and then things started to become dark even with this beautiful music still playing.” | Music unable to positively influence challenging experience |
|  | “I can remember thinking ‘this is beautiful music, why am I going to this dark place?’ It didn’t line up with what had gone on before, you know, that pattern. I just felt as if I was being manipulated, being duped almost.” | Dissonance, Sense of being manipulated |
|  | “A little conversely, the light music at one point took me to a place where I thought I was safe, and it became unsafe, and the music was playing a trick with me, you know, sort of giving me a false sense of security.” | Sense of being manipulated |
|  | I think really only that that luring beautiful woman’s voice that took me initially to a beautiful place, and then it took me to a really bad place. A *really* bad place.” | Transportation to “bad place” |
|  | “That was profound, because it was as though there was somebody orchestrating that, somebody manipulating that, you know, not in a good way.” | Sense of being manipulated |
| 19 | “I couldn’t connect with the music.” | Dissonance |

**Table 8 | Appreciated music styles and playlist features: music styles.** A list of all the themes and respective quotes by patients, that were present in the cluster “music styles”.

| **#** | **Quote** | **Theme** |
| --- | --- | --- |
| 1 | “I liked the piano pieces.” | Piano music |
|  | “A Spanish piece with a guitar and the singing, that was quite beautiful, I still remember that one.” | Ethnic or cultural music (Spanish), Guitar music, Vocal music |
|  | “Like I said there was a Spanish piece and there was a piece that sounded more African.” | Ethnic or cultural music (Spanish, African) |
| 2 | “It was a song that is quite special to me, there was the one in Spanish, I knew what it was saying. It’s a very popular song, especially for people of my age.” | Ethnic or cultural music (Spanish), Familiar music |
|  | “I think the one that I liked the most was the one in the beginning, sort of film-like music?” | Cinematic music |
|  | “There was some sort of activity going on and a suspense, and I did like that one.” | Cinematic music |
|  | “There was activity going on and at the same time some feeling of expected, which you would see in film, a soundtrack.” | Cinematic music |
| 3 | “At the end where there were more words, which was pretty good. I respect that maybe in the beginning I wouldn’t have appreciated that as much, when the drug was at its most intense.” | Vocal music |
|  | “There was an “African” song, where, I think they’re singing about “our father” or something? And it was just a whole bunch of voices most of the time in the song, which was pretty good.” | Ethnic or cultural music (African) |
|  | “There was one that just built up, [Greg Haines – Azure] was one example where in the beginning it just sounds like someone is just hitting sh*t randomly in his music room and then it all comes together in the end and blows your mind.” | Music with crescendo, powerful music |
|  | “Those aspects of it where it’s like, slightly… surprising. It just builds up, and with it your emotion goes with that and the happy feeling, and its like all connected.” | Music with surprise |
| 4 | “[The soothing music] brought up nice images.” | Soothing music |
|  | “The second time I sort of enjoyed crying to this music.” | Emotional music |
| 5 | “African-like music tapped into some deeper, more ancient part.” | Ethnic or cultural music (African) |
| 6(2) | "I knew lots of the music." | Familiar music |
|  | "I felt joy of the existence of Bach." | Classical or neo-classical music |
| 7 | “It began quite ambient-like, quite relaxing, which I felt was good. It calmed you in the experience.” | Ambient music |
|  | “Arabian music was quite strong.” | Ethnic or cultural music (Arabic), Powerful music |
|  | “With instrumental music [the story] is left to you.” | Instrumental music |
|  | “I preferred ambient music, and more broadly I preferred the music without lyrics.” | Music selection |
| 8 | "Quite liked the sort of more operatic one, with the lady that sang in Italian." | Vocal music (female), Choral or opera-like music |
|  | "Guitar playing, like a solo, I really liked that one." | Guitar music |
|  | "Some parts being more energetic, and more sort of driven." | Energetic music |
|  | "Other things being more quiet and subtle, and maybe encouraging reflection." | Calming music |
| 9 | “[Henry Gorecki – Symphony of sorrowful songs] starts off very nice chorally and then it goes off into a more kind of uncertain dark place and then comes back to the choral part again.” | Dynamic music, Choral or opera-like music |
|  | “[Greg Haines - Azure] I felt like I was sort of the highest I could get, it was like the absolute, the top of everything.” | Powerful music |
|  | “I thought ambient music would've resonated more with me than the rest” | Ambient music |
|  | “I'm not a massive classical music fan, but the classical pieces that were there worked really well.” | Classical or neoclassical music |
|  | “[Greg Haines - Azure] just builds and builds. You're holding on to an extent, you just kind of go up and you're like ‘ok, where am I? Can I go any further?’” | Music with crescendo |
| 10 | “It was more upbeat, so it was almost like it was arousing me from my slumber.” | Energetic music |
| 11 | “Indian music, where I was seeing an Indian temple.” | Ethnic or cultural music (Indian) |
|  | “For me what worked best in the sense of being the most facilitative to the trip, were the straight on classical pieces.” | Classical or neo-classical music |
|  | “I recognized a couple of pieces, those were very strong for me.” | Familiar music |
|  | “During that same Indian piece, that’s when I was dancing for Shiva and then I was Shiva.” | Ethnic or cultural music (Indian) |
|  | “Some Celtic music.” | Ethnic or cultural music (Celtic) |
|  | “That’s just what I normally respond to.” | Familiar music |
|  | “Pieces that had a solid drone to them drew me right in” | Drone music |
|  | “The drone, it was the chanted repetition of that, it was the layering of the other voices and the other instruments and the other syncopation and the rhythm as they would all come in and just build.” | Drone music, Vocal music (chanting), Music with regularity, Music with rhythm, Music with crescendo |
| 12 | “The violin music, it was stunningly beautiful.” | Violin music, Classical or neo-classical music |
|  | “[The violin music was] very beautiful. It’s one of my favourite kind of instruments.” | Violin music |
| 13 | “As the energy [in the music] kind of builds upward, it reaches a tension point.” | Music with crescendo |
|  | “But at the same time I kind of think that the other higher intensity stuff is probably necessary, I think, to get you along.” | Powerful music |
| 14 | "Primitive and deep “African” music, it wasn’t particularly pleasant in any way, but extraordinarily powerful” | Ethnic and cultural music (African) |
|  | “The Composer has stayed true to his or her own intuition or inspiration. Almost the antithesis to the Bach and the Brahms, which I felt were terribly contrived.” | Authentic music |
|  | “There was something in the regularity, but also the pattern, the pattern of it. I seem to be responding to the patterns of the sound.”” | Music with regularity |
| 15 | “One of the vocal tracks reminded me of one of Nusrat Fateh Ali Khan’s tracks, Yeni Yeniden, it’s so beautiful it just made me cry.” | Familiar music |
|  | [interviewer: were there any ones that you had a particular preference for?] “Yeah, the classical pieces.” | Classical or neo-classical music |
| 16 | “There were some [that I preferred], a man’s voice. The experience changed when there was a voice, from when it was just music.” | Vocal music (male) |
| 17 | “I heard the beginning of one of my favourite pieces of music. Beethoven’s Emperor Piano Concerto, the second movement.” | Familiar music, Classical or neo-classical music |
|  | “I preferred I suppose (chuckles) the sort of Classic FM stuff on the whole.” | Classical or neo-classical music |
|  | “I suppose church-type music, I think was responsible for enhancing the religious experience.” | Choral or opera-like music |
|  | “I think it was probably the great works of classical music that I preferred.” | Classical or neo-classical music |
|  | “Great works of music by great composers, it’s normally expected to sort of move you more.” | Classical or neo-classical music |
|  | “I think [classical music] was more moving.” | Classical or neo-classical music |
| 18 | “There was a woman’s beautiful voice.” | Vocal music (female) |
|  | “There was a specific moment when I felt as though I was being given birth to the universe, and I semi-recall there was this sort of crescendo of music at that point, that was a very strong point in the journey for me.” | Music with crescendo |
| 19 | “The Mexican song I thought was quite good.” | Ethnic or cultural music (Mexican) |
|  | “The Spanish guitar song was probably my favourite track out of all of them, with a man singing.” | Ethnic or cultural music (Spanish), Guitar music, Vocal music (male) |
|  | “It’s a classical track that was on both [playlists]. Probably a bit overused in the media, but an emotive piece of music.” | Classical or neo-classical music |
|  | “Because I play guitar, and I can’t play piano, I find guitar music more accessible.” | Guitar music, music with personal connection |
|  | “Like the kind of world music as well, it’s more interesting.” | Ethnic or cultural music |

**Table 9 | Appreciated music styles and playlist features: playlist design.** A list of all the themes and respective quotes by patients, that were present in the cluster “playlist features”.

| **#** | **Quote** | **Theme** |
| --- | --- | --- |
| 1 | “I liked that there was a lot of variety.” | Variety |
|  | “Gentle at the beginning, I think that that did help.” | Calming music in pre-onset |
|  | “Having the music in the background, it somehow just was helpful.” | Music presence |
|  | “I think that that it was comforting, knowing that the music was there.” | Music presence |
|  | “Variations in the degree of the music and the intensity of it. Some of it was more mellow, some of it more forceful.” | Variety |
|  | “[The music] was structured in line with the way the effects of the drug were.” | Music order |
|  | “I think that the music, because it was so varied I think it did kind of make you think a bit.” | Variety |
| 2 | “I think the one that I liked the most was the one in the beginning, sort of film-like music?” | Cinematic music in ascent-phase |
|  | “I could see that there was a great effort to put the music together in a way that it followed the experience as it was meant to be.” | Music order |
|  | “The fact that it was put together in that sequence made sense.” | Music order |
|  | “[The playlist was] made in a sort of logical order. It had some sort of beginning, halfway through, and then the end, it was logical, it was good.” | Music order |
| 3 | “There was quite a variance.” | Variety |
|  | “And most of it was good, because it was flipping good music.” | Music selection |
|  | “It was all good.” | Music selection |
|  | “I don’t know which one I’d say I prefer. I liked them.” | Music selection |
|  | “[The music] had their moments for each time of the dosage.” | Music order |
|  | “At the end where there were more words, which was pretty good. I respect that maybe in the beginning I wouldn’t have appreciated that as much, when the drug was at its most intense.” | Vocal music during return phase |
|  | “In the beginning part, the uh, the Greg Haines stuff was, pffff.. f*%#ing, in another planet!” | Music with crescendo during peak phase |
| 5 | “[I preferred] the changes in style.” | Variety |
|  | “I don’t know if I preferred any one above the other.” | Variety |
| 6 | "[The music] all worked really, really well." | Music selection |
|  | "Even bits that I didn't necessarily love, it all flowed." | Music selection |
| 7 | “Certainly the first time [the music had a greater influence], cause I think the second time you’re sort of familiar with the music.” | Unfamiliar music |
|  | “It began quite ambient-like, quite relaxing, which I felt was good. It calmed you in the experience.” | Calming music in pre-onset phase |
|  | “There was no uncertainty because of it, it didn’t create any anxiety or 'woah, what is this?!’” | Music selection |
|  | “[The songs] Superb they were without doubt you know.” | Music selection |
|  | “In terms of the musical choice I think it was absolutely superb.” | Music selection |
| 8 | "Well-composed sort of selection." | Music selection |
|  | "Well done, really well put together." | Music order |
|  | "I think I could kind of understand what each part was meant to evoke." | Understanding thoughts behind playlist design |
| 9 | “[Buffy Saint Marry - Up Where We Belong] You could tell that was quite cheesy, a bit of an anomaly in there, but no, it just worked.” | Music selection |
|  | “It all worked together really well.” | Music order |
|  | “The music worked really well, within the context of the rest of the music and that setting.” | Music order |
| 10 | “Whoever thought of putting that playlist together in the order that they did is a genius.” | Music order |
|  | “It was seamless- apart from the breaks. But after breaks they would put the headphones in and I would go straight back into that world.” | Music order, music presence |
|  | “Without it I would have gone mad.” | Music presence |
|  | “Certainly the experience would not have been a positive one were it not for that music.” | Music presence |
| 11 | “[Some songs] I connected immediately with.” | Music selection |
| 12 | “When there was a pause in the music it was really noticeable. It just felt that all the energy kind of drained out of the room, I really wanted the music back.” | Music presence |
|  | “There was one point, that was very beautiful.” | Music selection |
|  | “Wouldn’t have been as positive without the music, absolutely, you know. No, a hundred percent.” | Music presence |
|  | “I think it was designed that way.” | Music order |
| 13 | “And particularly once it starts to mellow out towards the end, you do feel that more relaxed.” | Calming music during return phase |
|  | “[I preferred] more towards the end of the session, I think, whenever it’s a bit more mellowed out. I kind of felt like a nicer place to be.” | Calming music during return phase |
|  | “The music is] definitely necessary.” | Music presence |
|  | “I wouldn’t see any major changes with the music.” | Music selection |
|  | “I think it was a good, a good layout.” | Music order |
| 14 | “I preferred Mendel’s playlist.” | Music selection |
| 15 | “An hour and a half before the end, when the music began to wind down, and it was at that point that I thought “oh yes, this is, this is much better. This is helping. Yeah, because I was slowly coming out. So yes, that was positive.” | Calming music during return phase |
|  | “One of the vocal tracks reminded me of one of Nusrat Fateh Ali Khan’s tracks, Yeni Yeniden, it’s so beautiful it just made me cry.” | Familiar music |
|  | [interviewer: were there any ones that you had a particular preference for?] “Yeah, the classical pieces.” | Classical or neo-classical music |
| 16 | “I get the impression that if the music wasn’t there it’d been a very different experience.” | Music presence |
|  | “There wasn’t any that I didn’t like. It felt like it all fitted the experience, and that everything was in the right order.” | Music selection, music order |
| 17 | “I was able to just put the headphones on and the mask on, and really enjoy it.” | Music selection |
|  | “When the music stopped it was a very spooky atmosphere.” | Music presence |
|  | “I think it was the beauty of the music, and the sort of uplifting nature.” | Music selection |
| 18 | “On the whole really lovely music, with obviously the dark music sometimes taking me to dark places, but I wasn’t really aware of that at the time, it just sort of happened.” | Music selection |

**Table 10 | Unappreciated music styles and playlist features: music styles.** A list of all the themes and respective quotes by patients, that were present in the cluster “music styles”.

| **Un-appreciated music styles and playlist features: music styles** | | |
| --- | --- | --- |
| **#** | **Quote** | **Theme** |
| 4 | “Very trippy music […] making me more scared when I had this difficult phase, that I was thinking ‘ah, I’m going crazy!’” | Trippy music |
| 5 | “It was all sad music.” | Sad music |
|  | “It’s all very serious music, there was no light. It was all really, serious, profound.” | Serious music |
| 7 | “I found lyrics confusing.” | Lyrics |
|  | “No interpretation, the story is being told to you… with lyrics, whereas with instrumental music it’s left to you.” | Lyrics |
| 11 | “I remember at some point saying “this music is really corny”, and there was one particular part where there was music going on that just reminded me of the ‘Sound of Music’, which our theatre in my little college town played for months, because that’s what the old folks in the town wanted to see, and it just had that sense of being that kind of kitsch almost.” | Cheesy music |
| 13 | “With a higher tempo everything feels a bit more tense.” | Music with high tempo |
| 14 | “I did struggle with the Brahms and the Bach.” | Classical or neo-classical music |
|  | “Almost the antithesis to the Bach and the the Brahms, which I felt were terribly contrived.” | Classical or neo-classical music |
|  | “I noticed I didn’t wanna hang around for the last hour, that version of Joe Cocker, ‘Love Lift Us Up’. I would consider that there was other music which is subtly more powerfully sort of joyous, rather than stating, claiming, ‘love will lift us up where we belong’.” | Music with lyrics, Cheesy music |
| 15 | “The piano pieces, irritated me. It was like little needles.” | Piano music |
|  | “I don’t know why, I found the vocal pieces so amusing.” | Vocal music |
| 16 | “I could remember hearing tracks that I’d heard before. I wasn’t unaware, you know, because I could remember music from the last time. And I could remember how I felt when I’d heard that track the first time, which was another thing that I found for the second one, which was ‘why is it not feeling the same?’” | Familiar music |
| 17 | “I didn’t really like the sort of world music stuff.” | Ethnic and cultural music |
|  | “There was some sort of Indian-style music, I think I found it a bit creepy at the time.” | Ethnic and cultural music (Indian) |
|  | “There was a sort of ambient musical sound which I found too non-specific.” | Ambient music |
|  | “I complained a bit about the ambient music because I thought it was it was too downbeat. It wasn’t sufficiently uplifting.” | Ambient music |
| 18 | “There was a kind of out-of-place piano, that was almost sort of ‘hunky-tunky’, but that just didn’t sort of fit really.” | Piano music |
|  | “[Henryk Gorecki – Symphony of sorrowful songs] It was a woman’s voice, it was a sort of operatic voice, and first it was beautiful because, you know, I was following it and it took me to a beautiful place. And then there was almost a shriek that started, and that that perhaps that shrieking, that really high pitched, almost reaching the end of that particular song, that was when things started to turn for me.” | Vocal music (female) |
| 19 | “I found the first [playlist] a bit of synthetic.” | “Synthetic” music |
|  | “Didn't like George Harrison or the Louis Armstrong. And there was that weird kind of cover [Buffy Saint Mary - Love Lifts Us Up]. It didn’t feel bad, it just felt a bit cheesy, a bit annoying. It was a bit like forcing a point.” | Cheesy music |
|  | “Even though there was the opera stuff, I can’t speak Latin, I couldn’t understand it” | Music with lyrics in foreign language |
|  | “I don't really understand opera.” | Music without a personal connection |
|  | “I think if you appreciate opera, Classic FM, you would have got something else out of it.” | Choral or opera-like music, Classical or neo-classical music |
|  | “Classical to me can be a bit inaccessible, a bit highbrow.” | Classical or neo-classical music |
|  | “Opera as well, because I don't understand the stories behind it.” | Choral or opera-like music, Music with lyrics in foreign language |

**Table 11 | Unappreciated music styles and playlist features: playlist design.** A list of all the themes and respective quotes by patients, that were present in the cluster “playlist features”.

| **#** | **Quote** | **Theme** |
| --- | --- | --- |
| 6 | "The majority of them are not my kind of music, I can't sit with that music, I have to leave the room." | Music selection |
|  | "It's music that I can't listen to, I find it irritating or agitating." | Music selection |
| 7 | “Second time, not as much [influence of the music].” | Familiar music |
|  | “I would finish with more sort of ambient music rather than to try and bring the lyrics in.” | Lyrics during return phase |
| 13 | “Some of the earlier stuff did get a bit tense at times.” | Intense music during ascent |
| 15 | “I did ask if I could have my own music, I would probably have chosen my own pieces.” | Preference for own music selection |
|  | “Less of the piano music.” | Piano music |
|  | “Maybe the pieces could be slightly shorter.” | Preference for shorter pieces |
| 17 | “When the music stopped and it was a very spooky atmosphere.” | Silences in playlist |
| 19 | “There was stuff in the second playlist I didn't like as well.” | Music selection |
|  | “Nothing I would have chosen if I wanted to relax.” | Preference for own music selection |
|  | “Like the kind of world music as well, it’s more interesting.” | Preference for more ethnic or cultural music |

**Music playlist (v1.2.) for psychedelic therapy sessions for depression with psilocybin**

The music playlist used in this study is displayed below. Please note that the playlist did not merely list the selected songs in the structure suggested below, but included a thoughtful mixing of volume, fade-ins, fade-outs, and moments of silence. Also note that following this study, an updated version of this playlist has been developed that incorporates insights from this study. I particular the return-phase has been replaced by calming ambient music. The latest playlist version can be found via **www.mendelkaelen.com**

**Time Track**

00:00:00 Stars of the Lid - Dungtitles (In A Major)

00:05:57 Stars of the Lid - Articulate Silences Part 1

00:11:20 Stars of the Lid - Articulate Silences Part 2

00:16:58 Stars of the Lid - Evil that never arrived

00:22:03 Harold Budd & John Fox - Sunlit Silhouettes

00:25:05 Harold Budd & John Fox - A Delicate Romance

00:32:36 Brain Eno & Harold Budd - Against the Sky

00:37:29 Brain Eno & Harold Budd - Lost in the Humming Air

00:41:43 Robert Rich & Lisa Moskow - Bija

00:52:24 Robert Rich – Sagrada Familia

00:56:15 Robert Rich – The spiral steps

*Silence (3 minutes)*

01:08:34 Henry Gorecki - Lento - Sostenuto Tranquillo Ma Cantabile

01:33:44 Maria Bayo & Sinfonica De Tenerife - Bailero

01:39:25 David Darling – Prayer for compassion

01:43:36 David Darling – Stones start spinning

01:47:53 Carlos Cipa – The Whole Truth

01:53:25 Greg Haines – 183 Times

02:02:36 Harold Budd & John Fox – Coming into focus

*Silence (30 seconds)*

02:08:06 Ludovico Einaudi - The Journey

02:10:43 Arvo Part – Da Pacem Domine

02:16:28 Max Richter – The Young Mariner

02:20:39 Max Richter – Diner and the ship of dreams

02:26:07 Henryk Gorecki - Lento e Largo Tranquillissimo

02:35:41 Greg Haines – Azure

*Silence (20 seconds)*

02:50:12 Otto A. Totland - Open

02:52:42 Otto A. Totland - Steps

02:54:34 Federico Albanese - Disclosed

*Silence (50 seconds)*

03:01:21 Dead Can Dance - Devorzhum

03:07:30 Robert Rich - Amrita (Water of Life)

03:13:56 Ólafur Arnalds & Alice Sara Ott - Verses

03:17:54 Ólafur Arnalds & Alice Sara Ott - Piano Sonata No.3 Largo

03:26:58 Ólafur Arnalds & Alice Sara Ott - Nocturne in C Sharp Minor

03:31:23 David Darling – Beautiful Life

03:33:30 David Darling – When we Forgive

03:37:35 Enya - sumiregusa

03:42:20 Jon Hassel, Ry Cooder & Ronu majumdar - Bay of Bengal

03:47:08 Jon Hassel, Ry Cooder & Ronu majumdar - River song

*Silence (25 seconds)*

04:12:55 Anugama - Shamanic dream

*Silence (20 seconds)*

04:24:26 Arvo part - Spiegel Im Spiegel

04:35:12 Nest - Stilness

04:40:49 Arve Henriksen - Glacier descent

04:48:17 Arve Henriksen - Opening Image

04:52:33 Arve Henriksen - Hambopolskavalsen

04:57:54 Daniel Namkhay - Um Bolero Galíctico

*Silence (15 seconds)*

05:04:05 Dead Can Dance - Nierika

*Silence (15 seconds)*

05:09:49 Brian McBride – Toil Theme Part 1,2& 3

05:17:31 Nils Frahm - Ambre

05:21:19 Nils Frahm - Tristana

*Silence (2.5 minutes)*

05:42:44 Mozart - Ave Verum Corpus

05:46:46 Mercedes Sosa - Gracias a la Vida

05:51:17 Ladysmith Black Mambazo - King of Kings

05:55:33 Buffy saint Mary - Up where we belong

06:00:10 Olafur Arnalds & Alice Sara Ott - Letters of a traveler

06:04:22 Stars of the Lid - Don't Bother They're Here
